# From the bit to the between: an autoethnography of friendship

**DOI:** 10.3389/fvets.2026.1865934

**Published:** 2026-09-01

**Authors:** Glen Cousquer

**Affiliations:** School of GeoSciences, University of Edinburgh, Edinburgh, United Kingdom

**Keywords:** attention, awareness, creativity, deep listening, dialogue, friendship, presence and forgiveness, working equine welfare

## Abstract

To develop any depth of understanding of friendships, there is a need to integrate holistic epistemological practices that respect the fluid relational and emergent nature of the between space where friends meet. This recognizes friendship as an experience always in process, never a thing or anything like a thing. Multispecies autoethnography is especially well suited to exploring such meetings, for it is concerned with issues of justice, power and freedom and therefore with addressing anthropocentricity. In this study, this qualitative methodology is presented and then developed to provide the reader with a rich ethnographic sense of the author's concerns about the quality of relationship made possible by the traditional bit and the need to prototype and demonstrate co-created alternatives. The work of Simone Weil on attention and Martin Buber on dialogue is emphasized throughout, as a theoretical framework for understanding the ways relationships founded on attentive listening and attunement can be nurtured in partnership. This example is full of misunderstandings and mismeetings, necessitating forgiveness on a regular basis and a commitment to show up, be present and relate. This study is not about friendships between conspecifics; it is about relationality and relational practice and how we practice working equine welfare, through the nurturing of humility and power literacy. This study offers insights and vocabulary that can help the reader develop an awareness-based systems change approach to equine welfare; one that involves listening to ourselves, learning to see, sense, and presence; one that, ultimately, allows equids and humans to nurture the between space where friendship can take root and grow. In doing so, this study provides insights into the possibilities of One Health justice.

## Introduction

Friendship, writes the philosopher David Whyte [(1), p. 71] “is a mirror to presence and a testament to forgiveness.” He continues, “all friendships of any length are based on a continued, mutual forgiveness.” [(1), p. 71]. This emphasis on continuance and mutuality captures the requirement for us to recognize friendship as relational, as an ongoing process that defies our Procrustean tendencies to define, delimit and control (2) and to deny that our lives are “already threaded through with other lives and deaths” (3). Any understanding of friendship and of forgiveness as a window into friendship needs to reckon with this threadedness, with our interbeing and intra-dependence (4–6). Friendship, as viewed through process philosophy, comes to life as we learn “to reconcile the tendency to flux with a tendency to stasis, change with some degree of permanence” [(2), p. 1240]. In reconciling what may appear incompatible, we find forgiveness and come to realize that “atonement is literally ‘at-one-ment”' [(2), p. 1240].

There was much to forgive and much forgiveness in my friendship with Bella, but it is the need for presence that this young mule taught me about, as we learnt to attend and listen to each other and co-presence the world. This is a multispecies autoethnographic (7) account of that friendship, of our meetings and mismeetings, of the co-sensing journey (8) and shadow work we undertook together, and of the lessons we learnt about presencing, forgiveness and therefore friendship within the *between*-space of the human-mule encounter (73). This *between*-space arises through co-creating *I-Thou* moments which allow friendship to emerge and ripen.

*I-Thou* moments consist not of two experiences dwelling distinctly in two persons but of a third dimension of “the between”, in which shared experiences enliven the *I-Thou* relationship. [(9), p. 24]

However, these meetings are momentary and elusive. They must be constantly nurtured if we are to realize this lively third dimension that Martin Buber encourages us to invest in, and that I believe grounds friendship. Friendship can be viewed as a liminal space in which modes of being and doing coalesce. It is in the quality of the being, however, that the relationship deepens, for how we are in the moment determines what can arise in the next. This being fully present is the elemental form of friendship, a form that has to be rediscovered again and again through attentional practice (10, 11, 30) as the friends continue to support each other's flourishing. The reason forgiveness is so crucial in friendship is that a soul friend (12) is present through our vulnerabilities, those dark nights of the soul when we are seeking to transcend our limitations and become a better friend to ourselves and others. It is in addressing the better part of ourselves that our friends can call us into living on the leading creative and elemental edges [(74), p. 58] of our possibilities. A friend can help us to engage with this edge work, to stay connected with the wealth of possibility we carry within and that can only arise through the opportunities we are presented for creativity, learning and growth [(13), p. xi]. If this is true of human-to-human friendships, it is also true of posthuman, that is to say more-than-human multispecies friendships, regardless of the power asymmetries, linguistic traps and lack of moral imagination [(14–16), pp. 35–36] [(75), p. 226] that have confounded research into friendship.

This study recognizes that friendship is “a moving frontier of understanding not only of the self and the other but also of a possible and as yet unlived, future.” [(1), p. 72]. This as-yet unimaginable future was very much part of the friendship formed *between* myself and Bella as we explored the possibilities of a future different from that typically offered the mules of her home valley (17). There were thus structural and intentional dimensions to this relationship, for we were co-investigators into how the welfare of working mules might be improved. The traditional practices, beliefs, equipment and forms of relationship and the ways these contribute to the assemblage that is mule welfare in the High Atlas of Morocco (17, 18) represent the contexts within which this study was undertaken and some of the sources of mismeetings and misunderstandings.

This friendship had its asymmetries and complexities, reflecting the situatedness of the project as it sought to test and challenge local traditional practices known to negatively impact mule welfare (4, 17, 19). This friendship was therefore messy (72) and hard to make sense of. It needed to be worked at. It necessitated a methodological approach sensitive to the manifestations of the particular, to intimacy and knowledge-making born of embodied presence and participatory wholeness (10), to the meaningful over the measurable (20), to relationship building and an attentiveness to the invisible processes unfolding in the *between*, that liminal space born of genuine dialogue where species meet (21, 22).

In this study, I recognize that my own study is fundamentally rooted in attentional practice, deep listening and awareness-based systems change. It is therefore entirely appropriate to draw on Kathryn Gillespie's call for multispecies autoethnography to be applied to understanding the complexities of relationships. She proposes the following:

Multispecies autoethnography as a way to attend to these relations and to bring together the generative methodological possibilities of multispecies fieldwork with autoethnography in order to reveal new ways of understanding and knowledge-making. Multispecies autoethnography can politicize intimate relationships of care, harm, power, and exclusion, and as such, can highlight aspects of human-animal relations that may be obscured in research that does not attend fully to the researcher's situatedness in a web of multispecies relations. [(7), p. 2100]

Gillespie sets out to unsettle anthropocentric thinking and to challenge exploitative relationships *between* humans and other species. My own research has similarly set out to render visible the impact of oppressive and exploitative practices, including those imposed by the use of the traditional bit and tethering (22–24); and to challenge these both directly and in more subtle ways through awareness-based systems change approaches (19).

In this study, a multispecies autoethnographic approach to improving working equine welfare is presented to demonstrate “what happens when we attend to and foreground relationships” that “privilege attentive listening and reciprocity” [(8), p. 191]. It is proposed that this allows knowing to arise through genuine dialogue and that such knowing is qualitatively different and highly relevant to any understanding of freedom and friendship. For Buber, turning to genuine meeting is the central relational event [(9), p. 162]. He asserts that “all real living is meeting … because genuine meeting requires an altogether different kind of attentiveness—a living relationship of whole person to whole person” [(9), p. 22]. As previously emphasized, this requires a holistic, embodied (25–28) experiential approach which focusses on the dimension of “the *between*”.

The between occurs when one turns to the other and enters into undivided relationships … “the between” is relational space ever and again reconstituted in our meetings with others and ever and again establishing genuine dialogue and genuine community. [(9), p. 78]

This approach holds much in common with Simone Weil's emphasis on recognizing the force of affliction and on paying attention. For Weil, the epistemological is the ethical for values and sensations are co-terminous [(11), p. 42]. How we read the world turns on our particular location (moral, social, political and economic) within the world, but this can be suspended if we understand attention as a negative effort, as a standing still, as a suspension that allows us to let go of our habitual ways of attending and seeing.

Suspension is the first move (76), a sacred pause [(29), pp. 49–71] that can be learnt. For Simone Weil, attention does not involve a contracting of our muscles but rather a turning toward another, a suspension of our thought, which leaves us “detached, empty and ready to be penetrated” [(30), p. 62]. Weil emphasizes how difficult it is to give one's attention to a sufferer:

The capacity to give one's attention to a sufferer is a very rare and difficult thing; it is almost a miracle. It is a miracle. Nearly all those who think they have this capacity do not possess it. [(30), p. 64].

Perhaps this is why Weil suffered so much with migraines. Without a mindful grounding practice, the effort feels impossible. Any ethnography of friendship must be able to establish a grounding in which our desires are canceled, and our blinding and bulimic self melts away [(11), p. 45]. This mirrors the call that Meister Eckhart and Rabbi Heschel have both articulated. For Eckhart, we need to be “empty in order to be fully present and fully mindful to the glory of existence” whilst for Heschel “standing still is a requisite in order to behold” [(79), p. 5]. A key aspect of the turning to the *I-Thou* that is fundamental to friendship is an ability to suspend, to pause, to empty oneself, to become available and to shift our source of attention from the head to the heart (10). This shift is central to the approach to awareness-based systems change that has become known as Theory U (31, 32) and has proven invaluable in understanding systems change in equine welfare (19).

This study sets out to explore the emergent possibility of human-equine friendship as is arises when we learn to make ourselves more fully available. It introduces the reader to the thinking of several key philosophers and the vocabulary they have developed to help us think about the relationships we co-create and the worlds we bring into being through our attentional practices. This study demonstrates how such thinking can be applied empirically as part of awareness-based systems change approaches to equine welfare (19, 80) and the role of multispecies autoethnography in both deepening and sharing insights. In doing so, it outlines the paradigm shift needed in equine welfare science to address inattention^1^ as the source of equine welfare problems, this shift requires us to appreciate that our habitual listening is problematic and that it is our habits of mind that keep us stuck. By developing our ability to shift where we listen from, it becomes possible to escape our perceptual habits, moving beyond the epistemic monocultures where valid knowledge is reduced to third-person, quantifiable, decontextualised data, to embrace a more holistic epistemic approach that recognizes the need for a wider range of perceptual capacities as sources of knowing.

I start first with an account of the qualitative research approach taken to explore how an attentional approach can help us understand friendship. This account emphasizes the onto-epistemological assumptions that must be appreciated to better understand multispecies autoethnographic study. I then present a selection of vignettes from the fieldwork undertaken with Bella to present facets of the multifaceted diamond that is human-equine friendship. I finish by discussing what this approach and these examples may help us to understand about freedom and friendship.

## Research approach

Autoethnographers draw on critical interpretive methodologies emphasizing the personal and biographical, the political, and the historical that render visible the oppressive structures of a culture [(77), p. 139] and point toward more socially just possibilities [(78), p. 596]. They do so by practicing autoethnography—a form of ethnography that foregrounds the self and recognizes the self as relational and situated, caught up in social, political and economic power systems [(7), p. 2099, (33)].

Autoethnography is a form of qualitative research, rooted in the feminist tradition, one that embraces subjectivity as a strength and challenges traditional notions of objectivity in research (34). It does so through its emphasis on positionality and reflexivity, positioning the self as both the site and source of knowledge production. This approach is particularly suited to the exploration of deeply personal and interpersonal lived experiences, generating insights whose wider social relevance might not emerge through more conventional methods (35). Through the interplay of narrative storytelling with critical analysis, appropriately supplemented by critical theory, autoethnography seeks to make meaningful, reflexive knowledge contributions that can advance both personal understanding and scholarly discourse (36).

As an approach, it is eminently suited to supporting researchers in their explorations of social, environmental and multispecies justice and transformative change. Such autoethnographic approaches are informed by critical theory and are termed critical autoethnographies, offering strategies to unmask, name and engage the powers (22, 37), curtailing abuses of power and privilege; challenging social injustices and inequities and changing dangerous beliefs and practices (38). As Adams [(38), p. 29] points out, this helps to “improve living conditions; promote resistance and transformation; and determine how to get along better, together, in ways that recognize and celebrate difference.”

For this study's desire to explore the role of presence and forgiveness in befriending and in negotiating freedoms and flourishing, it is helpful to say a little more about multispecies autoethnography as described by Gillespie (7) and Adams's [(38), p. 80] autoethnographic exploration of a call for forgiveness in education; a call that allows us to better live with the offenses we ourselves have committed and with the memories and scars we have acquired as a result of the intentional or unintentional actions of others, especially where power has been abused and injustice has been promoted.

## Multispecies autoethnography

Where multispecies ethnographers study the “contact zones where lines separating nature from culture have broken down, where encounters between *Homo sapiens* and other beings generate mutual ecologies and coproduced niches” [(39), p. 546], the study of multispecies autoethnographers is typically informed by a focus on and concerns about power and justice. In her seminal study on multispecies autoethnography, Kathryn Gillespie writes about the concerns that such approaches focus on:

For multispecies autoethnography, one of the core political questions at the heart of an anti-anthropocentric approach to the methodology is what this can do for other species to make a more just, gentle, and caring world for those who are so routinely subjects of normalized violence, instrumentalization, and dispossession. [(7), p. 2101]

She goes on to highlight that autoethnographic writing engages audiences beyond the academy by centring storytelling and articulating in compelling and moving ways the experiences and lives of the creatures who are the focus of the autoethnographer's research. She further emphasizes that autoethnographic writing has the ability to transform the researcher's own relationships with the more-than-human world.

Multispecies autoethnography challenges us to consider our own roles in the hegemonic violence that animals are subject to and how we can take responsibility for the ways we may have been actively complicit or implicated in harming others and ourselves (7). In practicing vulnerability and in exploring our own willingness to develop the necessary self-awareness and emotional literacy needed to engage with the emotionality of others (40), we can find ourselves developing our propensity for intimacy and our capacity for empathy and compassion, with and for ourselves and others (41, 42), including individuals of other species (43). This is very relevant to any exploration of friendship.

To appreciate its relevance, we must look more deeply into our relationship with flourishing, learning and growth, that is to say with transformational change. According to Scharmer and Pomeroy [(10), p. 9], transformative change is a relational quality that arises from a shift in our source of attention from empathic intersubjectivity to trans-subjectivity. It arises as boundaries dissolve, as we surrender into what is wanting to emerge. This is a form of birthing, a level of listening that connects us to the future possibilities that are dependent on us for their realization. Birthing is central to friendship, and to writing about friendship, for it is what makes possible renewal in each moment we are gifted. Drawing on Hannah Arendt, Lyndsey Stonebridge, in her wonderful study of freedom, writes of the significance of natality within our relationships:

A culture that worships birth is one that is essentially at ease with the idea of change, unpredictability and the new; not for change's sake (Arendt is not interested in monstrous political births) but because the human condition will always require new responses to a shifting and porous reality. This is why natality and action are so crucial to political plurality. We are not born to make the world in our image or bend it to our will, but to inhabit it with others, to act ‘in concert' with and between one another. [(82), p. 174].

Autoethnographic writing is therefore well suited to befriending, for this is a generative act, all the more so, where the possibilities one seeks are aligned with liberation and emancipation, not as endpoints but as relational practice. To understand this better, we need to appreciate how familiarity can be a “quiet death, an arrangement which permits the routine to continue without any new challenge or nourishment.” [(12), p. 122]. By staying curious about a friend, they can remain a mystery to us, and we can better embrace the adventure and the mystery of the friendship. As O'Donohue [(12), p. 123] writes: “To decide to view yourself as a complete stranger, someone who has just stepped ashore in your life, is a liberating exercise.” This is as true of the adventure of befriending ourselves as it is of befriending another, be they human or more-than-human. We have to care enough about these relationships to suspend the *Voice of Judgment* and exercise our curiosity (22, 32); such caring will also involve an element of forgiveness. Here is Gillespie on care in autoethnography:

Alongside a commitment to social justice, care is at the heart of autoethnography. It is often in and through relational practices of caring for, with, and about others and ourselves that the autoethnographic process is possible. In its careful attention to interpersonal interactions, emotion, embodiment, vulnerability, and precarity, autoethnography has the potential to contribute to moving toward a more caring society (96). Here, again, intimacy anchors autoethnography—the intimate relationships of care within which we are embedded with others are sources of knowledge- and world-making. [(7), p. 2102]

In conclusion then, the writing practice that is autoethnography offers us a source of knowledge born of many epistemological practices, including feminist care practices, somatic practice, mindfulness and experiential learning. They draw as much on intuition and imagination as sources of truth, as well as the overstated sources of truth provided by the left hemisphere (2). The practice of multispecies autoethnography deliberately sets out to decentre the human and center the non-human (7). For Vergara Kesada (44), this means slowing down and investing in relationship building and the establishment and study of intimate trusting relationships as a participant observer and then writing about these insights. Capturing these insights requires us to attend to our relational selves ethnographically. Writing about these insights challenges us to find words that speak to the complex assemblage of elements that come to constitute our relationships and that we can so easily remain oblivious to. In what follows, I start by writing about the traditional bit and how I came to attend to this device and then to shift my attention to the possibilities arising when dialogue is privileged and we invest in the between.

## Limitations

I fully acknowledge the interpretive and situated nature of multispecies autoethnography and the impossibility of fully accessing the mule's subjective experience. I further recognize that the mule does not have a right to reply and might well disagree with much of what I have said about, and tried to do to improve, her own life and that of her kind. That said, I am also acutely aware of the need for us to feel our way into the life world of others and to integrate multiple epistemologies (including somatic work, imagination, and intuition) into our sense-making and practices. This is about seeing beyond the teleological (the archetypal equine or mule) and the categories and projections we impose on others. It is about attending to the umwelt of each individual and caring about their experience of our shared experiences (20). It is also about refining ourselves as instruments of inquiry and learning to think from the whole rather than about the whole (10). Thus, it is important to know that this study recognizes that the validity of subjective (first person), intersubjective (second person) and trans-subjective (fourth person) epistemologies differs from that of objective epistemologies (third person).

## Attending to the bit

Simone Weil urges us to refuse to be an accomplice, to open our eyes and to attend to the force of affliction [(11), p. 39]. This is not easy, for it requires us to see the world as it is, to make present what is rendered absent (8), to see the suffering and hear the cries for help. Appeals for help represent calls to attend to the helpless, to their stories and to the multitude of ways these stories are overlooked and denied.

Attending to appeals for help is hard, however. For tourists and trekkers using pack mules in the international mountain tourism industry, it is easy to feel overwhelmed, to fail to see or understand. As “K” observed “it's not that we don't want to see … it's that there are so many things to see.” Despite this, tourists do care about the mule when they see her suffering; they just don't know how to attend to and interpret their cries for help (83). K concludes: “… the mules are not beggars, who deliberately get in your way and seek to attract your attention. They do not ask to be seen or to be helped.” But they do try to help themselves when their ability to accommodate the insensitivity of their handlers and riders is no longer enough, when too much is asked of them. Attending to such moments is important for it presences how and to what extent we absent ourselves from their suffering. This is the story of one such moment.

In March 2014, I witnessed a mule fall tragically over a cliff to her death, in front of a group of Norwegian walkers staying at a local hotel. The Norwegian guide accompanying the group^2^ provided the following description of the accident:

It was in the last week of March and our group of 15 members were walking up the path above the Kasbah along the track to Toubkal. When we were just under the road to Mzik we saw a man sitting on a mule standing on the road waiting for us to pass. The mule was standing very near the cliff side, when the first member in our group was around 20 meters from the mule, the mule suddenly, for no apparent reason, took a step aside and started to tumble down the cliff side. The man was still on the mule but fell off 10 meters below the road before the absolute vertical cliff. The mule was going tumbling on turn after turn and then over the vertical cliff and down to the riverside 30 meters below. There were [*sic*] not a sound from the mule during the fall.

We took care of the muledriver and gave him first aid, he was hurt but not so bad, no broken bones or arms. For us it was terrible to watch, some were crying and we were very touched by what happened in front of us.

Spontaneously we in our group collected a sum of 7.000 MAD to the family who had lost the important mule.

The group were badly traumatized by the experience. Stories crowd in, obscuring the reality for the mule. My own focus was on the mule, however, and I ran down to check that she had not survived the fall. Slowed by the difficult ground that lay between me and the waterfall below, it took me more than 10 min of scrambling to reach the mule. I was relieved to see a lifeless head peering up at me from the swirling waters of the Mizane river (Figures 1a, b), for her own sake and mine as I would have had no way of euthanizing her.

**Figure 1:**
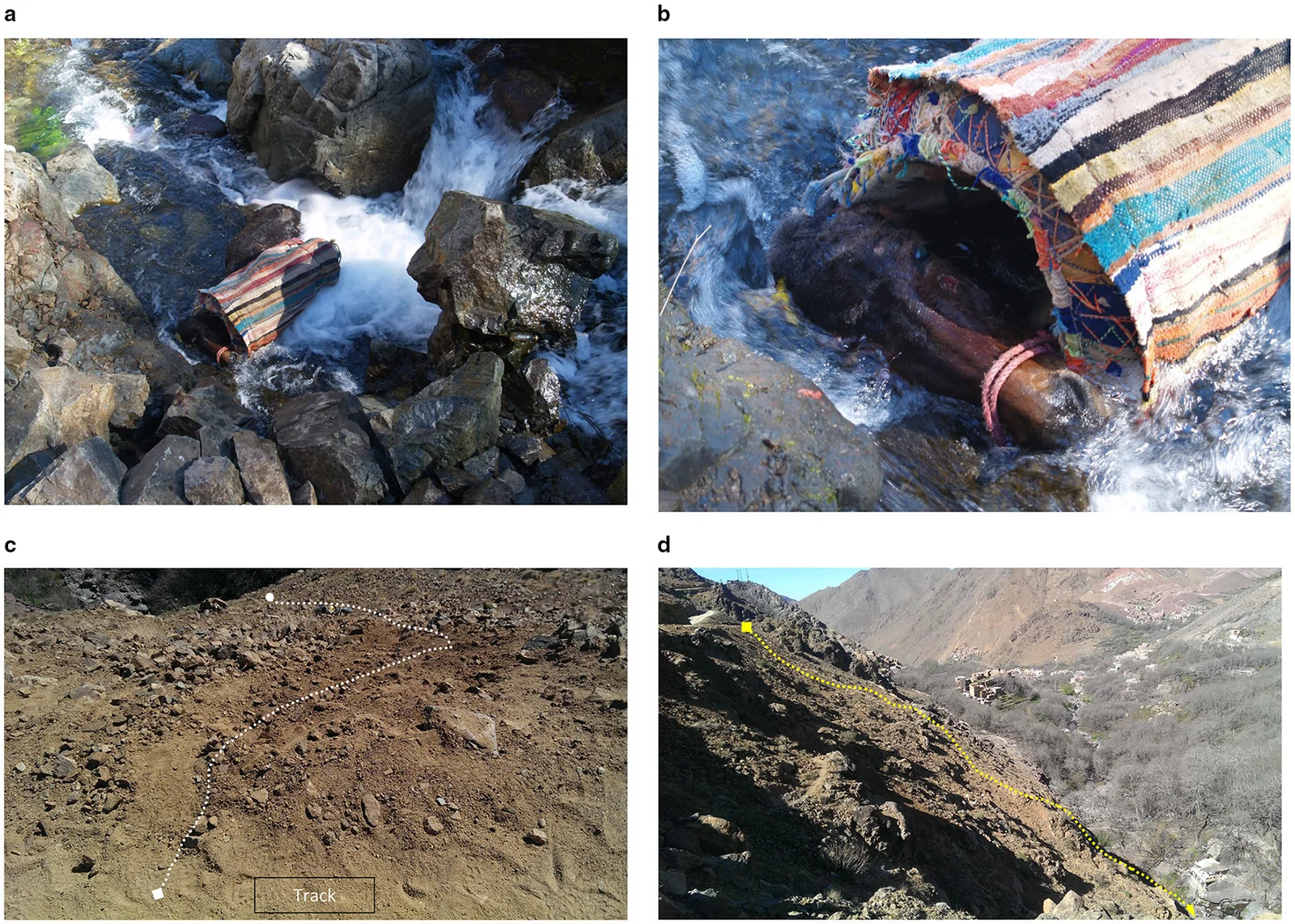
**(a, b)** The mule's fall ended in the river bed below the waterfalls above Imlil that countless thousands of tourists visit every summer. As ever, the pack saddle conceals, rendering invisible, the reality underneath: her abdomen had ruptured on impact, and her intestines were floating in the water. The cause of her fall is also hidden from those who cannot see beyond the human story to the mule story and the mule's lived reality. **(c, d)** The mule started backing away from the bit down the stony slope away from the edge of the track before throwing herself backwards and tumbling over the cliff side and down into the river below.

At the time, this accident was blamed on the mule stumbling or taking fright at the approach of the group. I was puzzled by the description of the mule “flipping over” provided by the local guide who was with the Norwegian group. I failed to see the significance of this detail; I could not understand why, on a broad track with the group still to emerge from the path that climbs up to the track, a mule should step over the edge and then throw herself backwards (Figures 1c, d). It was only as I came to understand the function of the traditional bit, the way it jams into the roof of the mouth and forces the mouth open and came to see how mules try to evade it by throwing their heads up that I started to see the actions of the rider and his abuse of the traditional bit in his mule's mouth as the likely cause of the accident.

Later, I discovered and came to understand how others had previously seen the abuse of the bit as the probable cause of such accidents. Cunninghame-Graham's [(45), p. 116] highly developed awareness of horses and horsemanship allows him to do just that in his account of a similar accident in Morocco.^3^ Such explanations, however, remain invisible and therefore deniable; it is far easier to blame the mule, to label her as difficult and a bit crazy. Attending well demands great awareness and ability. As McLean and McGreevy [(46), p. 189] point out, citing (47) even “trained judges have difficulty in detecting lightness” in the hands.

At the hotel, the generosity of the clients toward the mule owner meant he would now be able to purchase a replacement mule. No one, however, spared a thought for the mule or exercised their curiosity as to how this “accident” might have occurred. Therefore, we fail to see her attempts to escape, to help herself. We absent ourselves, closing our minds and hearts to her story, preferring another, more human, less Muley story, one in which the owner's loss and the tourist's concern that they may have caused the accident were both addressed. The traditional bit, the effect it has on the mule, the owner's reliance on a painful implement to communicate with, train and subjugate her, his lack of awareness of the alternatives and their relative merits, the lack of alternative equipment, education and training, none of these made it into the stories that were told around the dinner table that night.

The bit's absence from these stories is remarkable given its centrality! The material suffering and, in some cases, the deaths it mediates pass unrecognized as other protagonists and narratives take precedence. This does not have to be so. If we attend to the force mediating the affliction and what lies beyond, that is to say upstream of them—the bit, the hand, the mind, the human mind's power literacy and the relationship between man and mule—if we afford these concerns the power to command our attention, alternative stories can emerge.

Alternative narratives might choose to recognize the extent to which mules are brutalized and abused by local people with little or no training in animal communication and care, and the extent to which this is true. Communicating with mules by means of a traditional bit does not imply that local people mistreat their mules. There are too many counterexamples showing that there are many locals who do not abuse the bit, have even abandoned it, and enjoy a good relationship with their mules. It is, furthermore, worth noting that, over 100 years, [(84), p. 188] thought that Moroccans possessed “a community of feeling which I have never seen in any other land”. He argued that in Morocco the domestic animals and man understand each other better than in any other country he has visited. He bemoans how domestic animals are treated in the UK (the ridiculously cropped and docked hunters, the capitalistic-looking carriage horses), bemoaning too how animals have been turned into production machines back home (p. 189). He also wonders why a flock of sheep follows an Arab but is driven by a European. This highlights the industrialization of the human-non-human relationship that was occurring in the UK at the time of his writing and the relative freedom from such mechanization that can be found in Morocco. I suspect Cunninghame-Graham's observations say more about his disquiet with British practices than they do about Moroccan ones. There may even be a somewhat romanticized view of life in Morocco. Sheep and goats are certainly driven using stones in Morocco, as is emphasized by the proverb about a sheep jumping to avoid the stone thrown with pinpoint accuracy by their masters, only to guess wrong and be hit in the head.

Attending to the bit, to the material messages it transmits to the mule and fails to transmit to the muleteer, allows us to see that the bit is not an organ of loving attention. Attending to its brutal efficiency and insensitivity, we see how it, in turn, produces a brutal, insensitive handler who sees neither the suffering nor the damage done to their relationship, nor the need for an alternative. Attention here is to the I-in-Me and the *I-in-It* where the *It* is the mule, the bit and the study to be performed. In essence, the traditional bit makes it difficult to open to the other, to dialogue, and to establish a relationship based on mutual trust, respect and affection.

## Suspending and redirecting

As previously emphasized, suspension is the first move [(19), (31), pp. 35–36] (76), a sacred pause that can be learnt. For Weil [(30), p. 62], attentional study involves a suspension of our thought, which leaves us “detached, empty and ready to be penetrated” and a turning. This turning allows us to redirect our attention, to see more deeply [(31), pp. 148–149], more systemically, more imaginatively. Nonetheless, where does one redirect attention to?

The only alternative to the traditional bit available in the High Atlas of Morocco has, historically, been the curb bit provided through bit swapping clinics by a visiting animal welfare charity (48). Working within the limitations of a monthly visit to Imlil and without experts in equine behavior, groundwork, training, and riding, it is perhaps unsurprising that there has been a focus on bit swapping.

Bit swapping is a simple, unimaginative, one-size-fits-all solution that can deliver incremental improvements. It fails, however, to transform the situation. Replacing one bit for another assumes that the problem lies with the bit and that replacing a medieval device with a modern (more humane) device will solve “the problem”. The intervention can claim success without subjecting the underlying assumption(s) and diagnosis to critical evaluation. There is a failure to “look upstream” (49) to ascertain where the problem appears.

One of the charity's vets was led to understand from her team that “a mule that has had a traditional bit in its mouth for 5 or 6 years will not respond when a stainless-steel bit is used”.^4^ According to the same vet, “horsemanship skills take years to learn”. She did not think “vets … learnt about saddles or bits … this was something she had learnt as a rider”.^5^ This lack of awareness or “downloading” (32) coupled with the limited contact time their teams have with locals, makes it difficult for this charity to do more than provide alternative bits and a certain amount of advice on bitting and riding. Unfortunately, this also means that the alternative equipment supplied is not used effectively and is often deemed ineffective. One reason for this is the way the curb bit is mounted in the local bridle: a single cheek piece with sufficient movement at its extremity to cancel out the curb action. The bit therefore produces little in the way of poll pressure; its action is therefore negated, rendering it less effective. Another reason is that the bit cannot be used as a substitute for a lack of awareness and understanding of good mulemanship. Similarly, the bit does not address the assumption that a mule needs to be controlled rather than communicated with. Then, when the alternative is not readily available^6^ or is deemed ineffective, the status quo is perpetuated with owners falling back into their one truth, that of power-over and domination (22), thereby absenting themselves.

The assumption that the answer to the traditional bit is to replace it with another mechanical device thus obscures a host of alternative truths and narratives, including those offered by head collars and training in animal communication and handling. The focus on the bit appears to have made it difficult to redirect attention to the underlying relationship between the mule and her handler (Figure 2). Seeing with fresh eyes involves suspending patterns of the past and redirecting our attention to what lies upstream (32). Focussing on the bit rather than on the relationship means that the bit is changed rather than the fundamental relationship. Relationships founded on control rather than communication are not addressed; they are absent. It also means that those mules and owners who communicate well with their mules are not offered the training and equipment that would help them develop their practice to a higher level (presencing a latent potential). This would, of course, require a change in perspective and in strategy; sadly, individuals and organizations are often reluctant to evaluate and rethink how they operate.

**Figure 2:**
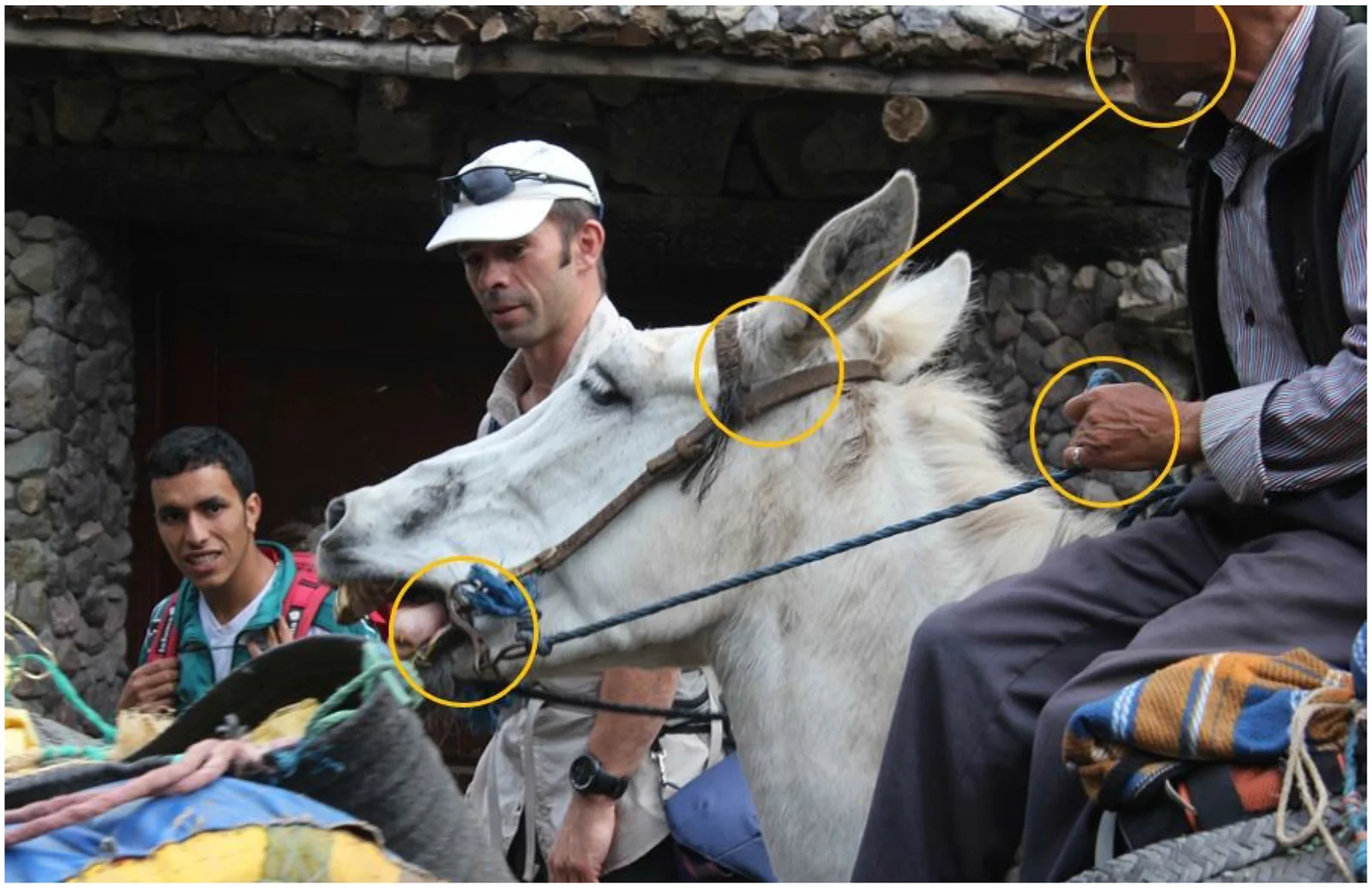
It is very easy for the attention of a concerned observer to focus on the mechanical device in the mule's mouth and perhaps the hand that directs that device. Directing attention to what lies upstream in the mind of the person controlling the mule via the bit brings knowledge and intentions into focus. Focussing on the mule and their experiences and, most importantly, the relational between space indicated by the orange line necessitates a radical change in approach.

My own awareness of the need to prototype alternatives led me to seek out opportunities to explore a move into the between, into dialogue, relationality and, arguably, friendship.

## From the bit to the between

If the bit is a monological device… If, in mediating and constituting the relationship, the bit brings forth an *It* (the objectified and exploited mule) and an *I-It:* the less-than-human muleteer… If “the development of the function of experiencing and using is mostly through a reduction in the power of relationship” [(85), p. 45]^7^ what happens when we attend to and foreground relationality? How, can we turn aside from the trodden path of *I-It* and explore alternative pathways that lead us to really^8^ know, care and take responsibility for, and perhaps even befriend, the mule? How can we develop our response-ability (4, 50)? What, in short, does it take for the*I*to refuse to affirm the *It* and, instead, affirm the *Thou* and open to friendship? Genuine relationships privilege attentive listening and reciprocity and are characterized by communication that is co-designed and co-created rather than imposed unilaterally. In shifting attention from the bit to the between, this next autoethnographic account explores the relationships and welfare that emerge when we know through genuine dialogue.

For Buber [(9), p. 162], turning to dialogue, to genuine meeting, is the central relational event. He asserts that “all real living is meeting … because genuine meeting requires an altogether different kind of attentiveness—a living relationship of whole person to whole person” [(9), p. 22]. How, if this is so, should we attend to the other if we are to genuinely meet, not just them but ourselves? The answer offered by Buber is to focus on the dimension of “the between”.

The between occurs when one turns to the other and enters into undivided relationships. … “the between” is relational space ever and again reconstituted in our meetings with others and ever and again establishing genuine dialogue and genuine community. [(9), p. 78]

This reminder of the essence of genuine meeting and dialogue is arguably essential to any understanding of friendship. Genuine meeting arises from a turning into relationship and is to be distinguished from the mismeetings born of wilful selfishness and individuality. It is grounded in a concern for the lived experience of the equine and the importance of positive, affiliative human–horse interactions that foster trust and invest positively in the relational “account” [(20), pp. 2–4].

Turning to friendship is born of movement; it crosses an inner threshold, one that recurs in each present moment, one that is defined by our quality of attention^9^ and presence in the moment; how we show up and the source from which we listen, think, and act defines, in turn, the quality of our relationship(s). I argue that it is in attending to the between that we can start to see how mule welfare is enacted (51) and where different versions of welfare originate from.

Attending to this movement and the embodied knowing that emerges when meeting and engaging a mule in dialogue is the focus of this chapter. By attending to the “between” in an embodied sense, I seek to develop an awareness of what it is to know, care and take responsibility for the mule, one's mule; in this case, my mule. I do this by attending to that which emerges through meeting, mutual reciprocity and dialogue (31, 32, 85), movement (52, 53), both physical and attitudinal, and through embodied awareness (25–28). I do this by “respecting” the between as only the eye that is the camera can:

I'm an eye. A mechanical eye. I, the machine, show you a world the way only I can see it. I free myself for today and forever from human immobility. I'm in constant movement. I approach and pull away from objects. I creep under them. I move alongside a running horse's mouth. I fall and rise with the falling and rising bodies. This is I, the machine, maneuvering in the chaotic movements, recording one movement after another... [(54), p. 17]

The eye thus attends to the I, the *I-It* and *I-Thou* and the myriad movements that allow us to know the mule.

The opportunity to explore how a mule could be trained and worked dialogically rather than monologically materialized when a “*Tachnit*”, or young mule, was purchased by a local establishment for 8,000 MAD,^10^ from a souk about 50 km to the west of Amizmiz and transported back to the village. She was approximately 4 years old, unnamed, untrained and with no scarring to her legs. She was a “blank canvas” (31, 32, 55).

This moment was the turning point at which my research shifted from understanding muleteers' practice and promoting awareness of welfare issues associated with the traditional bit, local tethering practices and overloading to exploring relational practice. In meeting this young mule, I was offered the opportunity to craft with her a different life from that she would have had. This was an opportunity to “prototype” (31) a different type of relationship. In this relationship, compulsion and the use of force were rejected outright from the start, forsaken too the unnatural and inhuman denial of movement, mobility and indeed agency [(52), p. 144] born of practices of confinement and restraint. There would be no tethers or hobbles, no *gamou* (bits), no *akouray* (sticks) or ear twisting; the relationship was to be stripped bare of these tools of domination and control. This decision introduced an element of uncertainty and therefore vulnerability [(86), pp. 52–54] [(9), pp. 33, 46, 167–169] into the relationship as we each let go of our assumptions and were present to each other, willing to be surprised and changed. We crossed the threshold together, and together we faced and overcame the challenges our journey threw at us. We learnt to attend to each other and were able to create a negotiated bond based on trust (20), understanding and genuine dialogue [(9, 85), p. 33].

This study provides an account of the meetings and mismeetings that marked our journey together. It is an account too of this mule's ability to frustrate me, to challenge me to listen both to her and to myself, to see her more fully, to exercise my curiosity and to change. It is an account of the embodied learning I experienced as she held up a mirror to me in which I could see my ability to be present, to forgive, and to earn her forgiveness and trust. It is also an account of how I learnt to “co-see”, “co-sense” and “co-create” (31, 32) with this young mule, where co-seeing involves opening the mind, co-sensing the heart and co-creating the will, providing us with access to the cognitive, affective and creative dimensions of relationality. Where co-seeing allows us to see the facts with more precision, it remains looking at. *Co-sensing* shifts us into attending with (10, 71), opening up an empathic dialogical relationality that allows us to relate empathically and uncover questions as pertinent as the questions asked by Ballam's donkey after he is unjustly hit three times by his unseeing and unfeeling master.

Then the Lord opened the donkey's mouth, and it said to Balaam,

What have I done to you to make you beat me these three times?^11^

These questions lead us to re-examine what we see-and-feel and do not see-and-feel: In my own case, I was allowed to question how my intentions aligned with my actions; this had implications for my inquiry into my own practice and that of others. I learnt to recognize that I needed help to suspend my own judgments and expectations and to see afresh. It was this realization that led me to seek out Ellen, whose ability to listen to and dialogue with equines has been honed into an art form.^12^ In November 2014, the two of us therefore became the three of us. Our journey together served as a lightning conductor, drawing out the knowledge, assumptions, beliefs and emotions of the mule's owners and handlers and opened discussions both at home and within the community on how a mule should be viewed, trained, and studied. This study built on earlier study to map the welfare concerns that mules working in the Moroccan mountain tourism industry are exposed to and set out to prototype alternative ways of relating and working that could be tested and shared with the wider industry.

The following account explores the U journey and

(i) how dialogue was initiated and genuine dialogue further cultivated^13^ through co-sensing journeys undertaken together.

(ii) Where dialogue was not established and the lessons emerging from such mis-meetings and the resulting need for forgiveness.
(iii) How we came to co-create a relationship and practices based on trust, understanding, mutuality, respect and dialogue and created a safe holding space in which to do this study that saw us move closer.
(iv) How embodying this relationship contributed to wider discussions about the basis for a good relationship between humans and mules.

## Initiating dialogue

Our first meeting was tentative and exploratory:

She was a little nervous when I approached … I was able to touch her shoulder without there being any reaction. I then ran my hand down her back and she gave a little buck when it ran past the withers. With some shaping work, I found that I was able to run my hand further and further down her back until she was happy for my hand to rest on her rump without there being any objection.

I explained the ‘shaping' concept … how we try to expand her comfort zone … that the least menacing point to approach her was the shoulder and that we needed to explore what she is comfortable with.^14^

Our meeting was two-sided and respectful. Berger [(54), p. 9] emphasizes the dialogical nature of encounter when he says that “the reciprocal nature of vision is more fundamental than that of spoken dialogue”. We exchanged looks, not words (Figure 3a). We looked and looked again. We “*respected*” the other. We did not presume to know each other, seeking instead to attend to and know the *Thou*. There was mutual reciprocity, presence without agendas—we simply made ourselves available, allowing things to unfold as we attended to each other by means of our proximity, curiosity, and openness. The embodiment of our mutuality allowed us to know whether and to what extent we accepted each other. Had I determined that our meeting was the moment to examine her teeth or remove the broken tether from her hind leg (Figure 3b), had I imposed myself on her, without asking her whether she chose to enter the relationship, things would have been different. To do so would have been to privilege the *I-It*. Instead, we chose *I-Thou* even if it only lasted until my hand failed to attend and she bucked. The quality of the “between” is thus honored.

**Figure 3:**
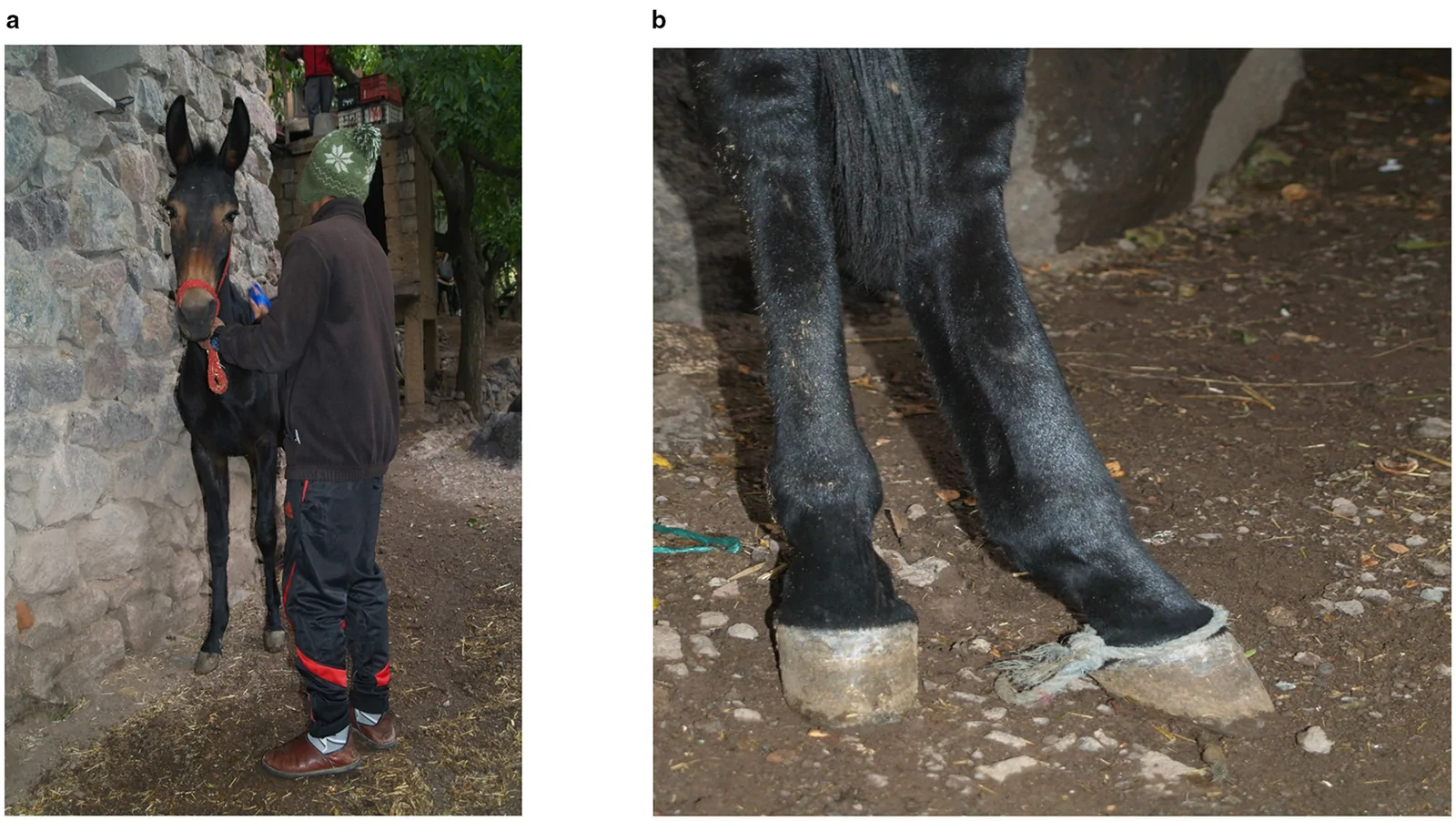
**(a)** First meeting: the young mule fixes her attention on me; I, in turn, attend to and photograph her. We are curious, interested in each other. I ask nothing of her other than watching her and her response as she is groomed. **(b)** A broken hobble on *Tachnit's* left hind leg bears witness to the common practice of tying front and back legs together to restrict the movement of young mules.

I emphasize the complete “letting go” [(31), pp. 184–185 and 399–401, (9), pp. 102–103] required for such a meeting. The act of turning away from personal wilfulness and toward relationship is crucial to the initiation of dialogue. It represents a surrender,^15^ a willingness to allow the future to emerge. As such, there is no pre-existing subjectivity. The meeting is not conceived here as an encounter between individuals whose actions and interactions—or “playful associations” [(56), p. 387]—can be assigned a multiplicity of intersubjective meanings that can then be compared. Entering into dialogue means letting go of such intentionality and attending to the other.

The next morning, we did not meet so well. My attention strayed from her onto other concerns; I thus tripped unwittingly on the threshold between *I-It* and *I-Thou*. I was absent, not present. This was a “mind the step” moment:

*Tachnit* had allowed herself to be led up a series of steps into the hotel garden and, once there, was keen to stay.

Getting her back down the steps was impossible!! No matter how much I tempted and cajoled her, she would not place more than her forefeet on the second from top step. She refused to budge any further. Any attempt to push or pull her (with a rope round her bottom) just made things worse. One of the staff tried lifting her feet and placing them lower down—and had to be told that this would never work as she had not decided to move. I started to think I had been too bold and would now be seen not to have the answers. I did not want to leave them with this problem.^16^

She was not supposed to be in the garden. The garden is all flowers and water features. It is for the guests. I was aware we should not be there. She did not share that awareness or understanding; neither did she share my desire to return her back to the cold and grassless underworld from which she had come. I struggled to let go of my personal wilfulness and turn fully to her. I had some awareness of the problem and of the need to rethink the situation, but I did not know how to be present. Operating in the world of *I-It*, I therefore sought a logical solution:

How was I going to change her mind? Then the answer came—she needed to follow a friend. **K** acted on my request and asked **D** to bring his own mule to the bottom of the steps. Seeing her there, the young mule almost pelted down the steps. I had a job keeping her to a walk. (See text footnote **16**)

This set the pattern for our learning together. The mule said “No!”, she refused, she ignored what I thought were quite reasonable requests, she was awkward, difficult, and stubborn.

No!... No?... Those are my words. Those are the judgments that flow from a human who, when confronted with a mule, does not provide the mule with the space, time and opportunity to express themselves. My “requests” were human requests or, as Game [(27), p. 1] argues, “human-centered questions”.

This mule's refusal challenged me to let go of what I thought “reasonable”, to let go of my agenda and see things from her perspective. I needed to hear her “Yes” where I heard a “No”. She was inviting me not to force her over the threshold but to ask her to step over it with me. She was asking me to take her needs, feelings and preferences into account, asking me to listen not to my narrative(s) but to hers. She was challenging me to step out of my bubble to allow myself to see and witness her as an essential self [(31), pp. 179–180]. In addition, in challenging me to attend more fully to her, she was helping develop my awareness of my intentions and of how my actions aligned with them. I was thus challenged to listen to her questions rather than focus on my own. As Merrifield [(57), p. 30] argues of donkeys, they challenge you to be patient, to listen, to “speak *correctement*”, helping us appreciate that (p. 40) we don't see things as they are but as we are. How I was seeing determined what I saw [(58), p. 63].

Being present with *Tachnit* meant, among other things, respecting her, not fearing her. We therefore had to study what we needed to achieve a listening space … the kind in which there are no distractions, and we could dialogue; the kind of space too, perhaps, where we would be able to critically reflect on our practice through being rather than doing.

I am not suggesting that we engaged in critical reflection together but that she was there “in the arena” with me; she was not a spectator in the stands but someone whose face was marked by dust and sweat, much as mine.^17^ The trust, respect and understanding we achieved were thus won in the moments we were present to each other, won through dialogue and not through the use of force and abuse of power.

## Mismeetings

Mismeetings were common features of the co-sensing journeys undertaken in the early days. Learning from them was important in order to meet genuinely: When visiting her first thing in the morning, I realized that it was too much to ask to walk into her stable and put her head collar on. She was not comfortable with this, turning her bum toward me. I let go, and I redirected my attention to her. She was thirsty and, having been locked indoors for between 8 and 12 h, without water, light or company, was keen to get out and drink. Aligning our intentions thus came to mean opening the door, waiting for her to come to me before putting on her head collar and walking with her to the water trough. I wanted her to recognize that it was comfortable to be with me [(59), p. 28] and to choose to be with me.

I was open to her and aware that when her needs were not respected, she would make her feelings known to those willing and able to listen:

The young mule was still locked in her stable. I let her out and the first thing she did was to go for a long drink from the water trough. She was clearly very thirsty! I can't blame her given that she was probably as much as 18 hours without access to water. She was also a little bit grumpy and barged me when, after a few pieces, I refused to feed her the rest of the apple I had in my hand. There was even a small buck. At the time, I was a little annoyed but on reflection I can see that it was her pent-up frustration.

…

When the staff finally turned up, I asked them why the young mule had been left in the dark and without water all morning. One of them let her out of her small compound and she spent the next ten minutes or so charging around like a lunatic. She appeared to be really enjoying the freedom and space that had been denied to her all morning.^18^

In North Africa, equines have long been required to wait patiently for their master and are typically not watered until the afternoon [(60), pp. 152, 178], thereby developing their ability to withstand the hardships imposed by their master. That is not to say, however, that this is entirely born of cruelty and brutality; we must remember that the harsh life of the desert nomad (87, 88) that gives rise to “les mœurs du désert” is full of contradictions and that the love Arabs bear for the horse reflects these [(60), pp. 95–107].^19^ Whilst their practices may be the product of circumstance and necessity, I argue that they are also born of singularity—of a hierarchical way of ordering the world that leaves little room for tyranny to be challenged and reimagined.^20^ Generative dialogue, by contrast, offers new possibilities, for neither the objectives nor the outcomes are predetermined. My focus here is therefore on the (e)quality of the between. Intentions in action thus emerge out of ongoing interaction (89). According to Fantasia et al. [(61), p. 14], the minimum requirement is that interactors share an interactional space and cooperate in sense-making, forming goals and purposes together whilst interacting.

Mismeetings taught me to anticipate (and imagine^21^) what might upset her. This was needed to avoid potentially difficult or even dangerous situations, but also because such mismeetings were not productive; they reinforced existing positions and implied a turning inwards, whereas meeting implies a turning away from wilfulness toward the other; this willingness must be learnt by both parties. Mismeetings also taught me to forgive myself for my own mistakes and to nurture a growth mindset that allowed mismeetings to become learning opportunities.

The drive to secure and consume food could give rise to wilful self-interest for *Tachnit*. On one occasion she was persuaded back into her enclosure with an offering of oats^22^:

When she eventually came down the steps, I placed the bucket on the floor for her. She started bucking, catching me on the back of the fingers, where they were resting over my upper right leg. We … retrieved the bucket with a long-handled rubber scraper—from the safety of the steps. I did not get angry with her or feel anger toward her. I just pointed out that this would be another area in which we would need to invest time and effort, so she feels comfortable with us being around her food.^23^

This represents a mismeeting, a turning away from me and toward food; it was something I needed to guard against. The daily challenges thrown up by her socialization and training thus helped develop our awareness of how she attended, of her availability. She, in turn, became aware of when we were available to her. This emphasis on our attitudes toward each other is part of an embodied approach to cooperation that recognizes that the enactive perspective involves “cooperative interaction before communicative abilities are achieved” [(61), p. 874].

Focussing on the other and not on objectives taught me to give her time to decide to take on certain challenges. This involved me reading her carefully so that I was aware of when I was pushing her into the stretch zone and potentially asking too much of her. Self-awareness was also required to identify when I had turned away from her and become preoccupied with my own feelings and objectives. Awareness of the turn away and of the turn toward are crucial, providing insights into one's attitude. Experiencing this “double act of turning” [(9), pp. 156–177],^24^ this inner movement and its outer manifestations and consequences helped me acquire awareness of the subtle difference between *I-It* and *I-Thou* ways of relating and communicating.

The hairpin bend in the path down to the village was just such a challenge. She did not like it and was unwilling to follow the bend through. She was challenged to navigate the turn; we were challenged to understand her and patiently rethink and represent the challenge. Staying present to each other was difficult; however, when failure lured us into absencing. Finding ourselves stuck, like some broken-down lorry blocking the High Street, I felt my stress levels rising as I became conscious of the pile of people we were holding up. I was to experience absenting many times, only coming to recognize it as such much later:

This morning I took the young mule down to the village. It was a disastrous experience. She refused to move forwards all over the place and I lost my patience with her several times. She often took her food and then stopped moving forwards altogether. It was most frustrating. I tried all the usual tricks including turning her, reversing her and tempting her with various items and pulling on her lead rope. I was soon running out of bread and having to resort to brown walnut leaves. I tried pulling her upper lip, then her lower lip and sometimes this seemed to produce some forward motion but it was very short-lived. I was very conscious of everyone going past and seeing her ‘misbehaving'. I was starting to question why I was not allowing those who wanted to shoo her on (or hit her for that matter) from behind do so.^25^

I was not attending to *Tachnit*. I was not present. My attention was on moving forwards, on objectives, my objectives. I also failed to see that she was attending not to me but to the food in my pocket.^26^ Absencing is a very real problem, especially when you do not become aware of it in time to interrupt what you are doing and collect yourself:

Shortly after the hairpin bend, she again stopped and refused to move forwards. … After some five minutes or so of frustration, a young girl … came up behind her. I signaled to [her]to stop and go over the wall but the young girl came on and received a small kick from the mule. She was caught somewhere around her waistline and started crying. She was, however, able to walk and walked away.

One of the men who had been watching then told me that I should have been paying attention. I told him that it was because I had been paying attention that I had told her and signaled to her to move to the side. He said that she was Berber and did not understand this. This just added to my frustration for the fact that she did not understand my words should not have prevented her from understanding the hand signal or from knowing that you don't just walk behind a mule. I felt sad and guilty and angry that this had happened. (See text footnote **26**)

Had I recognized that we were not dialoguing, that we had turned away from each other, I could have interrupted or discontinued the walk and avoided the incident. This was the low point of our relationship, a veritable mismeeting.

I turned the mule around and then headed back uphill. This took a long time and a lot of pulling. I was trying to allow the release on the lead rope as she moved forwards to reward her. She was stopping and starting. She was taking all the food and biting my fingers. She was being awkward and I confess that I ended up punching her a few times when she refused to move at all. (See text footnote **28**)

I felt ashamed to have allowed myself to get annoyed with her to the point that I hit her. She too had allowed herself to kick me at times, and we both had to learn that this was unnecessary, that this was a dead end on our journey together, one that was not worth exploring.

Gradually, my awareness of how my world view and perceptions affected how I interacted with her grew as I reflected on my ability to be patient, to let go of objectives that I had formed an attachment to, to break down super objectives into attainable, less daunting steps [(59), p. 28]. I became more curious and imaginative, more able to go with the flow. Confronting my own impatience and inflexibility took time but eventually led to an attitudinal shift. Understanding that the objective of any interaction with her was not immovable but fluid meant that our interactions became much more like a free-flowing dance in which we constantly either attended to each other or failed to do so. For me this meant “letting go” of any attachment I might have to specific possibilities, to agendas. By opening myself up to the *Thou*, by yielding to the moment, to what was ready to emerge when we both privileged the mutual presence that arose between us, I started to experience and learn about a different *I*. I embodied a new way of knowing and caring. I was transformed.

Relation is mutual. My Thou affects me, as I affect it. We are molded by our pupils and built by our works. The “bad” man lightly touched by the holy primary word, becomes one who reveals. How we are educated by children and by animals. [(85), pp. 15–16]

This transformation through mutual presence was evident during an attempted river q27crossing after heavy rain. I had walked *Tachnit* up the steep mule path to her home, crossing a series of streams that had appeared as the water cascaded from the mountainside above. Opposite the village of Aremd, the river was swollen and running fast. Despite us having never crossed the river when it was a trickle, I asked her to cross. The objective could have been to get to the other side, in which case, failure was very much an option. There was, however, no predefined objective; they were emergent. She yielded to the pressure on her lead rope and crossed the first part of the river only to refuse to go any further. My urgings did not persuade her to budge an inch.^27^ I therefore returned to the section we had just crossed and asked her to reverse this. She refused. Still in my own map, I tried moving her side to side; I tried tempting her with pocket treats; I tried leaning into the lead rope so that she felt the pressure come on through her head collar. She refused, as only mules can, to re-enter the water and cross. Staring possible defeat in the face, I experienced an attitudinal shift, seeing not failure (an argument born of the imposition and rejection of my answer) but options (a conversation born of a question and the possibility of a co-created answer). We headed upstream and found a series of smaller crossing points. We entered the water together. My feet fully submerged, I walked her down the stream to the point where she had refused to cross, and there exited the stream.

This was an end we chose together, that came when we let go of what we thought we wanted and attended to each other, allowing something to emerge from the between. Genuine relationship cannot, however, be willed; something else is needed and this, according to Buber is grace.

… the *Thou* meets me through the effective grace of reciprocal acts of compassion. Action and surrender, will and relational grace generate the interactive immediacy of meeting. For Buber, grace is not a theological term but the spontaneously undetermined presence of mutuality, which cannot be activated by will alone. [(9), p. 22]

Letting go can mean many things when seeking to study with a mule. In my case, I learnt to relinquish objectives and any tendency I might have not to listen. I had to learn to be present or at least to be aware of when judgment, fear and pride prevented me from being present-in-the-moment and from seeing the whole reality that lay before me. Turning toward and attending to the other leads naturally to a letting go of the desire to control situations. It was, in recognizing this, that I came to realize that I had to let go of control and make myself vulnerable; it was in this space that trust and dialogue emerged. Countering fear and pride, letting go of control are thus essential parts of the co-sensing journey that lead to generative dialogue and co-creation.

This journey was difficult. I made mistakes. I sometimes went too fast and asked too much, largely because I felt the need to prove that a better way of relating was possible and because I wanted this better way to be part of this mule's future. Also, when this happened, I had to recognize my mistake. In other words, I had to learn humility. In this next extract from my field notes, I build on a successful moment and try to go further, without checking in first with the young mule:

I repeatedly brought her back legs up and then asked Hassan to do the same. To my amazement, he actually succeeded without any problems. I then went too far, letting go of her lead rope whilst taking a back foot on my knee. She moved forwards and kicked me; one kick catching me in the abdomen!^28^

The kick was unwelcome and potentially dangerous; the reality, however, was that I stopped listening. In this situation, I needed to let go of any resentment toward the mule, forgive her and admit that I had failed to listen to the subtle clues she had given me that I was asking too much and deciding to focus my attention on listening more attentively rather than on neutralizing the risk of a kick left me better able to dialogue. Then how? How was I present to *Tachnit*, how did I come to know her better and come to care for her wellbeing? What happens when judgment is suspended, and attention redirected (31)? What do we look at (52), to what do we redirect our attention?

Bortoft (90) and Scharmer (31) suggest that redirecting out attention from the obvious issue to what lies behind involves going upstream. What did I find upstream? Among other things, I found my attitude(s), intention(s) and emotion(s) and my ability to attend to these and act mindfully, aligning my actions with my intention to forge a respectful, dialogical relationship.

I experienced strong emotional responses, including frustration, fear and anger. Marks [(59), pp. 44–47] emphasizes the importance of “attitude” and the need to analyze where anger comes from to stay calm. I also found judgments (she's stubborn, dangerous and can't be trusted), cynicism (she's never going to learn) and fear (I'm going to get hurt, shown up, proven wrong) or a focus on objectives (it's going really well, we're both doing well, let's keep going^29^) getting in the way of listening. Also attending to listening, getting curious about it, and listening first actively and then obediently can initiate turning [(9), p. 172].

Refocussing from the bit to the between thus leads us to attend to the quality of communication and relationship. This shift in attention is profoundly significant for attending to the between; it leads us to ask whether that communication is characterized by reciprocal mutuality and dialogue or one-sided singularity and monolog. This, in turn, gives rise to an awareness of who is present, the *I* born of *I-It* or *I-Thou*.

The account provided so far of some of my early exchanges with *Tachnit* provides a flavor of our mismeetings and the glimpses I gained of what it might feel like to meet her genuinely. What I was only dimly aware of was that the*I*present at any one moment in time was dependent on an inner movement, one that profoundly influenced not just the nature of the encounter but the future that could emerge from that encounter depending on whether it was a genuine meeting or a mismeeting.

An essential part of Theory U's approach involves turning the camera back on yourself [(31), p. 15].^30^ Ellen's awareness and presence, our use of photographs and video, allowed us to do exactly that. She pointed the camera and captured our interactions. By slowing down the interaction, by freezing it, we started to see elements of the interaction that had eluded us. Listening was made present as was its absence. The moments in time had both a past (attitude, intentions and emotions) and a future (consequences). These were not lent but evidenced; these photographs were therefore meaningful [(62), p. 91] for they allowed us to understand how the past fed into the present moment, giving rise to the future that unfolded after the photograph was taken. This meant that the between could genuinely become a focus of attention. We found ourselves discussing the various ways *Tachnit* and I attended (or failed to attend) to each other. Gradually, we edged toward an awareness of whether we were absenting ourselves or truly present. An awareness of absence and of presence is bound up with mismeetings and meetings. The photographer's attention was on communication and by focussing on the quality of attention, it was possible to develop our awareness of the absence or presence of mutuality and dialogue. The next section explores how this was achieved.

## Prototyping a relationship founded on trust and dialogue

Prototyping a relationship with *Tachnit* founded on trust and dialogue meant that we had to learn to meet, to feel safe together, and to attend to each other as one whole person to another. Opening to and being open with means letting go of any hidden agendas and simply being present in the moment.

Genuine meeting embodies directness and wholeness. By directness Buber means immediacy, presence without agendas. [(9), p. 22]

For working equines, especially in sport, their economic value “encourages a foreshortening of training in many instances to the detriment of the animal's welfare” [(46), pp. 187–188]. Genuine meeting, however, values the whole person, not their utility. In forsaking the tools that foreshorten training and impose control, I was privileging dialogue and uncertainty for, in relinquishing control, the outcome was uncertain and therefore meaningful [(62), p. 91].

I do not pretend that the account that follows captures all the mystery of our meetings. I am, however, humbly focussing on certain aspects that I believe to be significant and meaningful. These are naming, choosing to move closer, and embodied attunement. These are each, in their own way, *I-Thou* movements.

## Naming

Honoring the uniqueness of the person necessitates seeing them as individuals. According to Finch (63), names are, at least in contemporary Western Society, a marker both of individuality and kinship. For Fudge (64, 65), however, one of the barriers to animal biographies (and arguably therefore to individuality) is the absence of a name.^31^ Opening to *Tachnit*, honoring her story, meant seeing her as more than a foal. She needed a name. There was, however, a problem: mules are called, in Berber, either foal (*Tachnit*) or mule (*Aserdoun* if male, *Taserdount* if female). There are exceptions, but these are very rare and usually instigated by foreign visitors. Brown [(66), p. 5] named the two mules that accompanied him on his 90-day traverse of the Atlas, Taza and Tamri, after his starting and finishing points. They “would become distinct personalities in the weeks ahead... and by far our biggest and most constant concern,” he remarks. (p. 18). To understand why, in North African culture, despite their importance,^32^ they are not named, it is helpful to refer to the Abd el-Kader and Dumas [(60), pp. 164–165]:

Despite all the bonds that unite man and horse, despite the solidarity born of familiarity, interests and religion, the Muslim will never give to his horse a human name. These have been born by saints: It would be a great sin, a sacrilege, to apply them to an animal, even if he were the noblest of all animals. We give names only to the most illustrious horses...

A list of suitable names follows, describing qualities (Victorious, Patient, Pure, Swift), colors (Tar, Ruby, Night) or other animals (Gazelle, Dove, Grasshopper) before the telling and final line on the matter: “We give, more or less, the same names to slaves” (p. 164).

It was therefore hardly surprising that the call for names met with little response from the local population who could not be persuaded to see beyond *Tachnit* and *Taserdount*. A call for names was issued, giving rise to a list, from which the young mule's handlers and owners chose “Bella”. Then Bella, she became.

Is it necessary to name the other to meet genuinely? No, for such meetings do not involve words; they involve attention, feeling, and understanding. Words, lest we forget it, divide. What is called for when dialoguing is respect of the synechistic ontology (67) born of integrity (91, 92), and the wholeness implicit in *I-Thou* encounters [(93), p. 95] [(9), pp. 99–101]. A name is, I believe, essential, however, when speaking and writing of her and of our meetings. In speaking of Bella, I intend audiences to understand that I speak not of any young mule but of Bella. In introducing her name so late in this autoethnography, I also invite the reader to feel the unease that knowing her as “foal” can give rise to. I ask you to attend to how we meet the other. In short, I ask you to experience the shift as we move away from attending as *I-It* and attend as *I-Thou*.

## Choosing to move closer

Dialogical wholeness involves both surrender and action, for one has both to choose to enter into relationship with and be reciprocally chosen [(9), p. 22]. This implies choosing to move closer, overcoming judgment, cynicism and fear (31, 32), surrendering into curiosity, compassion and courage (20, 22). It also implies embracing the uncertainty and mystery that genuine meeting and dialogue give rise to.

Moving closer together implies feeling safe and trusting. Establishing mutual trust is a communicative endeavor. By attending to the between, by caring about how Bella felt and, perhaps most importantly, by trusting and respecting her, we came to know and care for each other.

Attending to Bella meant letting go of my fears and my need to control the situation. The same was true in reverse. We both avoided threats and threatening situations. We both wanted safety, a safe space in which we could dialogue. How was this achieved? Creating a safe space in which a relationship can be developed creatively, with each partner suspending their own inner voices and attending to the other, opens up a world of possibility.^33^ Being present to each other must recognize this as art, for it arises as the product of two creative intelligences open to each other and to that which might emerge as they interact. For Scharmer [(31), pp. 187–188], a holding space is “consciously created through a process of context sharing, storytelling and deep listening”; it is “when deep calls unto deep” [(9), p. 78]. It took time for Bella and I to understand what that safe space looked and felt like. We studied in her stable, in the enclosure that led from the stable, on the flat area of ground above the stable, in the garden and on the paths above the village. In doing so, we explored the psychological dimensions of a safe space as well as the more obvious physical ones. It was, very simply, a space, a narrow ridge [(94), p. 204] in which we could give our full attention and truly listen to each other. In this space, outside distractions were eliminated, and both of us let go of our fears. For both of us, this space can perhaps best be summarized by the triangle formed by the mule's body and my two arms (Figures 4a, b).

**Figure 4:**
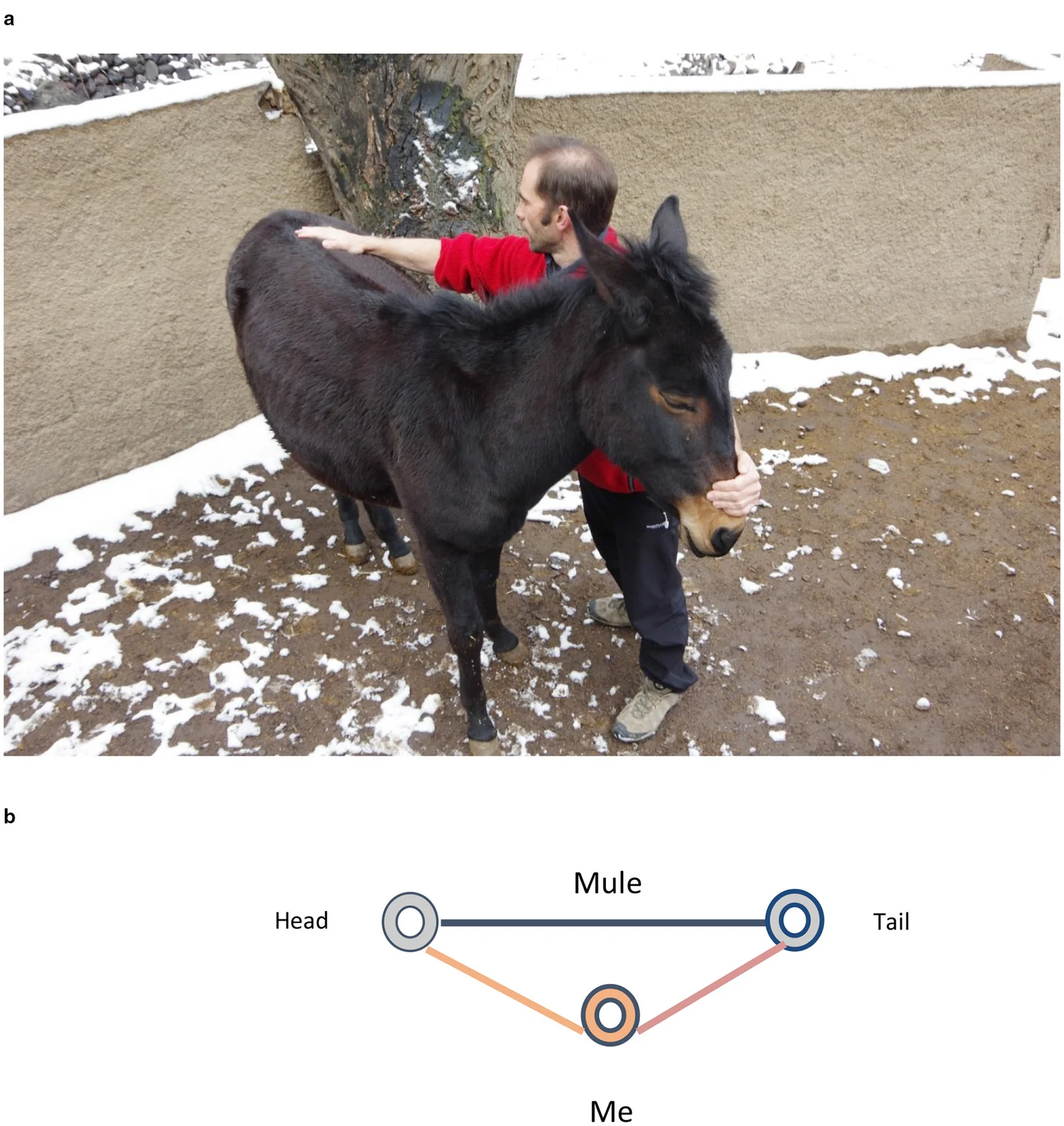
**(a)** A safe triangle is formed by the mule and the arms of the handler. The hands lie gently over the pelvis and nose, allowing for two-way communication. Both individuals are thus in dialogue; both feel safe and are open to the other. **(b)** A safe triangle feels safe because both parties feel unthreatened and calm. They are in contact, sustained physical contact; dialogue is thus sustained and intentions clarified.

Initially, the stairs down into Bella's enclosure provided me with safe access into her compound. I could sit on the steps and engage her curiosity. She was very food-oriented, and this was used to ask her to come to me. Asking her to stand calmly was rewarded with a piece of apple. She came to associate this request for calmness with me saying “SShhhhh” and standing up straight to her. The next step consisted of stepping down from the safety of the steps and again asking her to come to me. From this position, it was easy to step into her shoulder and establish the safe triangle.

During the initial approach, my attention is on reading her; Bella's on reading me. She is constantly reading her environment for threats, and I needed to step into her World map and learn to see from her perspective. This involves listening to her, feeling whether she is relaxed, watching her ears, facial expression, and tail, evaluating her behavior and constantly asking if she is ok with you being there. It also involves telling her that you are calm—dropping your shoulders, opening your chest, talking to her, moving slowly and steadily, embodying calmness. This mutuality is dialogical, providing us with moment-by-moment opportunities to trust each other. That trust was embodied; we experienced and came to know what it feels like to trust each other.

Establishing a safe space drew on an exploration of our respective comfort zones. In doing so, countless thresholds were crossed. Then each time we returned from venturing over the threshold and into our respective stretch zones, we had to find ways of integrating our new awareness into the practices—the way(s) of being together—that we were co-creating. The growth of our respective comfort zones is something we thus embodied not individually, but collectively, we grew together.

I felt comfortable at her shoulder and, in this position, could turn her head toward me and turn her rear end away from me. This safe and strong position reassures the handler and creates the circumstances for listening, which, in turn, communicates itself to the mule. In this position, the age-old fear of being kicked by a mule is suspended and can come to be forgotten altogether as trust develops. Confidence is thus embodied; it is felt and experienced and can be cultivated. It communicates itself to the mule, for she starts to see consistency and comes to understand clear messages. It also communicates itself to those watching and helps undermine and challenge their own fears and distrust, an outcome that I was very keen to deliver as a way to prototype and demonstrate what is possible and to render less credible the Moroccan saying that warns people not to trust a mule as, even dead, they will kick you with their shoes (68).^34^

Choosing to move closer to each other implies freedom of choice. It implies the give and take of a request, the possibility of a refusal. There is no room for command and the certainty of obedience. Dialogue implies uncertainty. The judicious use of good positioning coupled with my increasing ability to attend to Bella meant that I could focus on her and on what lay between us. Initially, our communication was mediated by a head collar and lead rope, gentle handling devices that allowed me to ask her to turn her head and bend her neck toward me. This helped initiate a turning circle that, crucially, allows the safe place to be preserved or re-established. I stayed at her shoulder, where neither of us felt threatened. For me, this is one of the essential movements to practice with a mule if one wants to be able to calm, slow and stop her. Calming here is crucial, for, in creating a space for judgments, suspicion and fear to be suspended, it was possible for Bella and I to experience calmness together, to engage curiously and learn to trust each other.

On the trail, I could step back to her shoulder and ask her to turn her head and bend her neck (Figures 5a, b). This movement was practiced until it became second nature to us both. Accompanied by a calming voice and a little reassurance, it helps re-establish that safe space.

**Figure 5:**
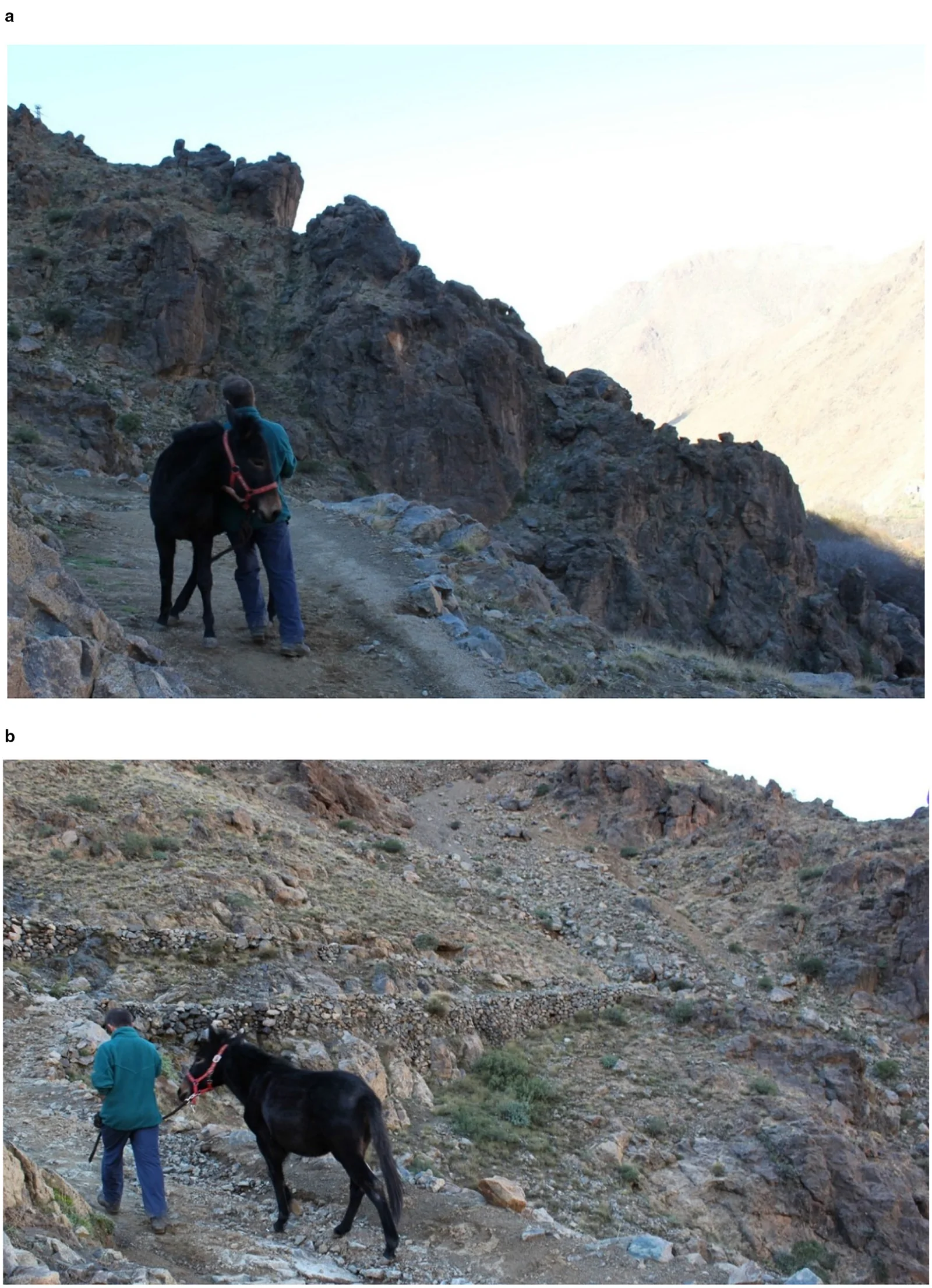
**(a)** On the trail, Bella and I practice turning a tight circle together. This serves as a means of re-establishing connection and dialogue, for she is asked to listen to me. Once the understanding is established, we can walk on. **(b)** Note the slack lead rope held across my body and the attention Bella is paying to me.

The descent … was attempted for the first time. She was a … handful at times and at one point, tried to bite the fleece at the back of my neck and jump onto me. I had to have stern words with her and turn her tightly. She was perhaps being playful and did not realize that it was a potentially dangerous situation. I was surprisingly unconcerned about the edge, however, and knew that she would turn easily enough.^35^

## Moving with

The “shaping” study [(59), pp. 206–207] undertaken meant that the threshold for a kick^36^ continued to rise as awareness, trust and understanding are developed. We could both sense how comfortable we felt in each other's proximity. We could feel each other, the muscle tension, the resistance, when one or the other moves, and learn to respond appropriately. In the same way that Fantasia et al. [(61), p. 6] argue of the cooperative process that intentions and goals become “manifest in speakers' behavior, shaped and adjusted” as interactions unfold, I argue that, as fears subside, as our own inner voices quieten, a listening space emerges in which one hears, sees and can come to know the other with outcomes arising from this encounter. I further concur with Fantasia et al. (61) that this does not require mutual understanding of each other's interest or purpose regarding the shared action.

To better feel her, to develop deeper awareness of how she was feeling, I found myself abandoning the head collar and getting ever closer to her until there was nothing between us; nothing and everything. We were one; our relationship was “of persons mutually and reciprocally united” [(9), p. 80]. We learnt to move together gradually as we came to know and trust each other. When offering her food, I would pass the food from my offering hand to the other hand, thus bringing her head and neck across my body. This could be done with a hand on the head collar, then with no hand on the head collar but with contact maintained via the lead rope, then with no head collar.

Turning was developed further as a safe, trusting knowing was established. When undertaking groundwork with her, I initially stood off from her shoulder, with a hand on her lead rein or head collar and another over her pelvis. To progress this, I took to standing with my back against her, her head cupped in my arm. I could feel her and how she was feeling. I could turn my whole body and, with it, her head and neck (Figures 6a, b). Indeed, when I leaned back, she started to turn around me, and this became a dependable way of turning her. She thus learnt to feel comfortable wrapped around me and wrapping herself around me. This is known as feeling safe, as mutual trust, as oneness. Turning and circling were thus achieved (of which more later), turning not just around each other but toward each other. Turning “away from a self-reflexive monolog … and toward the wordless depths of genuine *I-Thou* dialogue [(9), p. 159].

**Figure 6:**
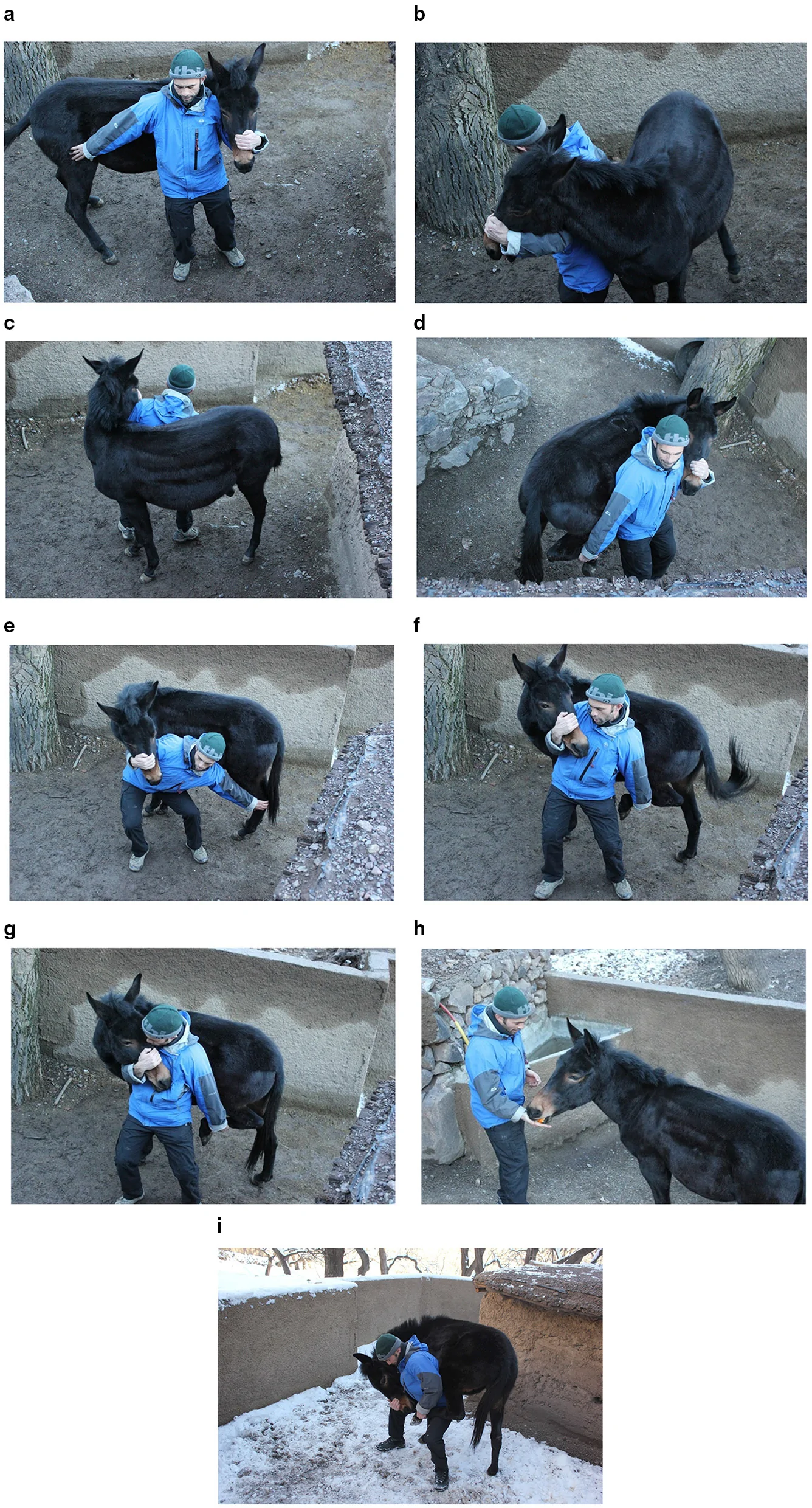
**(a)** After stepping into her shoulder, a cupped hand can ask her to bring her head round; she is wrapping herself around me. **(b)** We move together. I lean back and slightly away from her head to initiate a turn that, if pursued, becomes a circle. **(c)** With the mule's head turned and my own knees bent, a little of my weight can be pushed backwards as I ask her to give me her hind leg. She has to offer it, though. **(d)** If she does not want to and resists (or kicks), I am going too fast. We are thus listening to each other. **(e)** Listening to each other employs all the senses. Above, I can feel that she is relaxed; I know she is listening to me and is curious. **(f)** I can sense her tail hanging calmly, and when it does flick briefly when the foot is raised, I know from her state of mind that it is momentary. **(g)** The tail settles. She is calm and relaxed as I am. We are connected in that moment; there is I-Thou. **(h)** It could be argued that the reward for her is being listened to and that the orange peel is unnecessary. It is all, however, part of the mutual relationship we forged together. **(i)** Wordless, embodied dialogue was developed to the point where Bella would allow me to touch her nose with her hind foot and would remain calm and accepting throughout.

In this position, my free hand was available to run toward and down her hind leg, exploring how accepting she was of this. This step-by-step exploration of our respective comfort zones is a trust exercise, a form of dialogue; it repeats the question, are you ok with this? If she feels relaxed and accepting, I allow my hand to gently carry on down her back leg to her foot. It is only when she is happy with my hand resting there that I lean back into her and, in doing so, shift her weight slightly onto the contralateral limb. A gentle lift brings the foot up, and this is held briefly with the hand relaxed (Figures 6c–g). I learnt to recognize when to stop, when I am asking too much, relinquishing any desire to keep pushing and asking. I thank her (Figure 6h), and maybe she thanks me. Therefore, the shaping continues. Gradually we build up to the point where Bella is so relaxed that I can touch her nose with her hind foot (Figure 6i) and let go of her head whilst still holding her hind foot. This is significant because, at this point, I am vulnerable: If I have not been listening to her, if I have misjudged how relaxed she is, I can get kicked. It is not a question of courage though for, if I have to ask that question, it is because I am listening to myself (absenting myself)^37^ when I should be listening to the mule. This level of awareness of the other is born of hours of communion, communication, and genuine community [(9), pp. 73–95].

When standing facing her and asking her to turn her head and give me her back leg (Figures 7a–c), the same attentiveness and awareness is called for. In this case, letting go of the head allows me to run my hand down her neck and over her back before, again, asking for the back foot. Complete trust is required of her as she is free to respond as she likes (Figure 7d); this demands presence. As trust builds, we discover a surprising ability to be curious; when judgment is suspended, curiosity floods in and I can crouch beside her, making myself vulnerable and ask her to lift her front leg. We are each thus making ourselves vulnerable (Figures 8a–d). Soon I can pick her feet up and pick them out without there being any need to hold her head, leaving her free to exercise her curiosity and playfulness (Figure 8e). This is when I know that her real character is starting to show itself. In such moments, we are present to each other.

**Figure 7:**
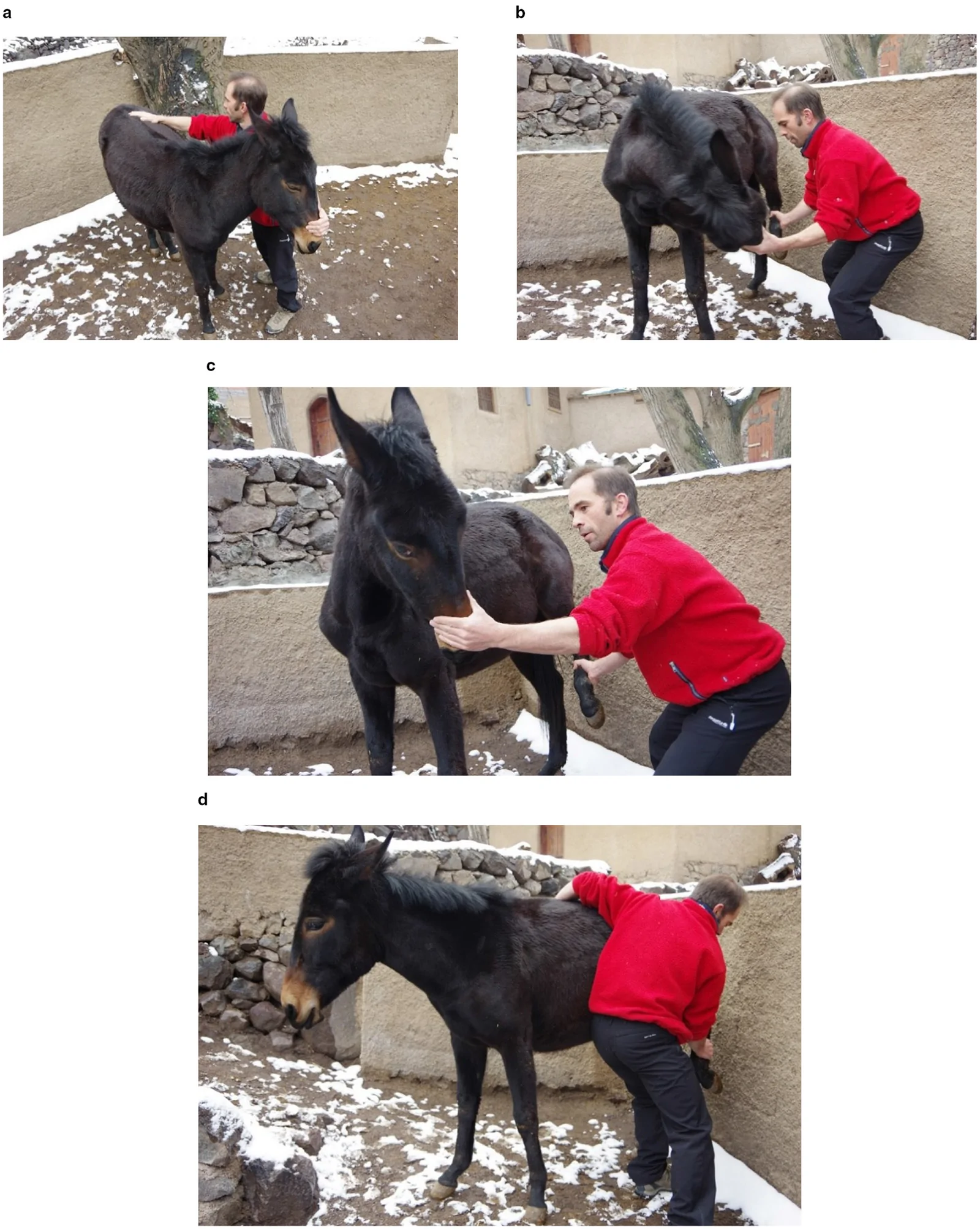
**(a)** Bella stands calmly whilst I run my hand over her back. **(b)** In a matter of seconds, I am touching, then picking up her back leg, whilst showing her what I am doing. **(c, d)** I then let go of her head and move my hand down her back, taking her back foot on my knee. Note how she is listening to me when I have released her head. Her ears are turned towards me, our bodies and embodied movements moving in concert.

**Figure 8:**
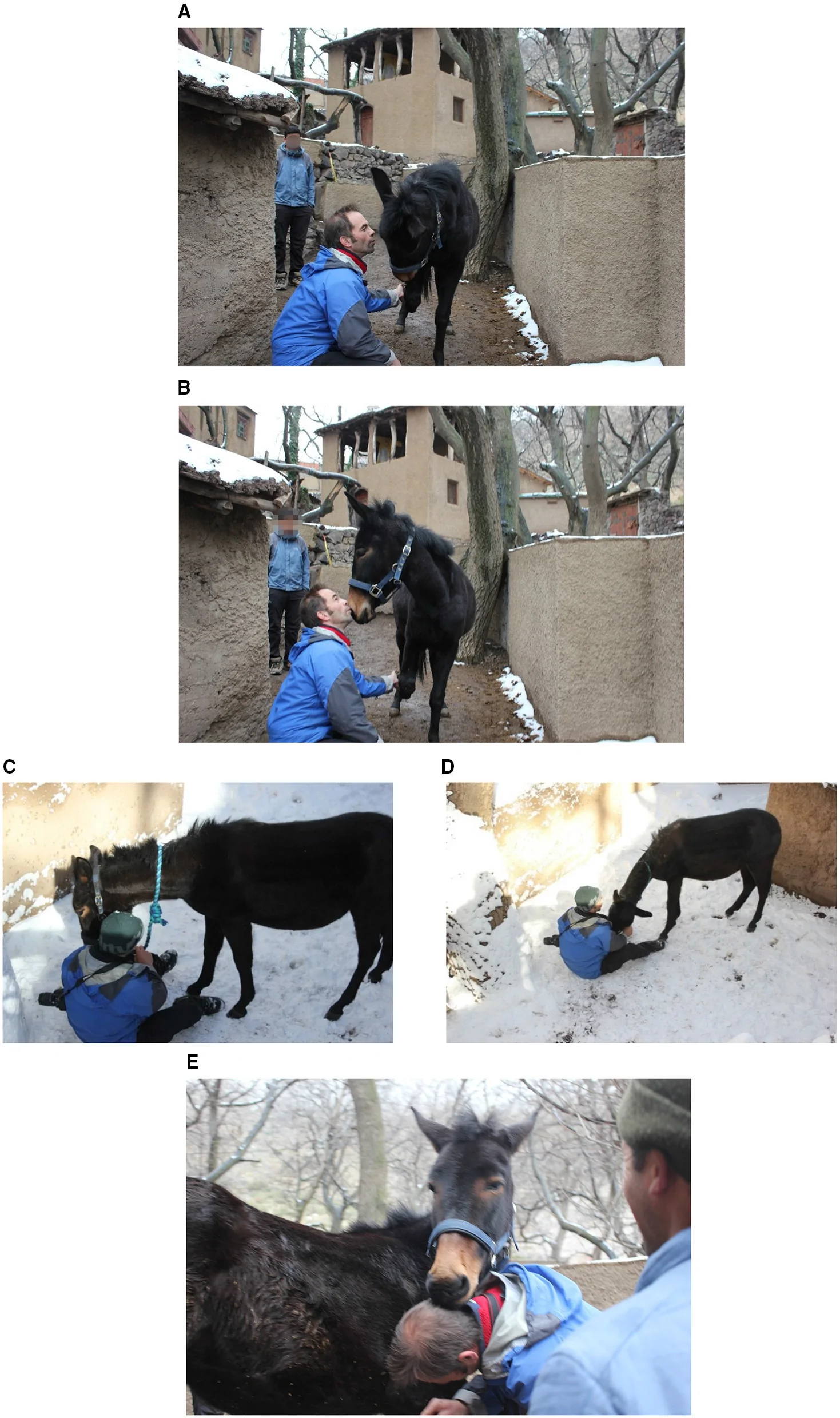
**(a)** Bella appears curious to see what I am doing with her leg. **(b)** She then presents her face and invites me to blow into her nose. **(c)** Making oneself vulnerable involves surrendering one's defenses. This requires that trust be established through dialogue. **(d)** Here, I have chosen to sit on the floor and engage Bella in conversation. She is calm and curious, playful even. **(e)** Here, I am cleaning out one of the mule's front feet whilst she appears happy to explore what I might think of her nibbling the hair at the back of my head. This playfulness is born of trust, curiosity and mutuality.

Berger [(54), p. 8] argues that “to touch something is to situate oneself in relation to it” and (p. 9) that “the faculty of touch is like a static, limited form of sight”. In learning to feed Bella, I open not just my palm but myself to her. I trust her to take food gently from my hand; she trusts me to give it. Surrender and action [(9), p. 22]. My hand meets her lips. We meet. She makes use of the long sensory vibrissae on her muzzle and lips. Calmness, stillness, and presence allow us to do this. Gone are the abrupt movements born of fear. Bella develops confidence in the consistency of my approach. I can then go further. When I ask her to take my hand into her mouth, I do so step-by-step. I ask her if she is ok with my hand over her lips, then establish that she is ok with a finger inside the lip. I then run that finger over her gums. Each time she confirms she is ok. There comes a point when I can place that finger trustingly between her incisors, knowing she will not bite. I can also offer up my hand and/or arm for her to lick and nibble; we even establish that she is ok with my arm across her mouth.

## Conclusion

This autoethnographic account has explored some of the dimensions of human-equine friendship as they become manifest through attentional dialogical practice. The dimensions that come through strongly in this account are those of mutuality, vulnerability, forgiveness and trust. In writing about this relationship and the many ways that I sought to spare Bella the harsh treatment and injustices that await so many mules, I have tried to make sense of the ethnographic practices that made this possible as I developed my own relationship with myself and with Bella. This has involved thick description (69) of experiences where seeing and sensing were privileged. I recognize that seeing, sensing, and presencing come before words (54) and that these attentional practices establish our place in the world. “We explain our place in the world with words, but words can never undo the fact that we are surrounded by it” [(54), p. 7]. In seeking to understand friendship, we must resist any temptation to analyze it, for this is born of apprehending rather than comprehending (2). Thus, our writing must speak to the whole experience, must try to respect the glimpses into a phenomenon that is relational all the way through. As Berger (54) writes, the relation between what we see and what we know is never settled, for we can always see further into ourselves, our ways of seeing and sensing and acting and therefore our relationships and our friendships. Writing about our multispecies befriending practices supports us in understanding and nurturing these. The mule's ability to hold up a mirror to our intentions and the extent to which we are fully present and integrated (19, 70) is remarkable and represents an invitation to soul-searching and humility. David Whyte's proposal that friendship is a mirror to presence and a testament to forgiveness is therefore worth revisiting. For me, I must also learn to forgive myself for the times I struggled to be present, to be a good friend, and for not having the wherewithal to prepare Bella for a life in which she would not always be treated with dignity and respect.

For the field of equine welfare, this study demonstrates the value of multispecies autoethnography and awareness-based systems change as methodologies for reimagining and re-configuring who we are in relation to equines and the freedoms and relationships we offer them. As the equine welfare and veterinary sciences seek to explore the potential for systems thinking (80) and awareness-based systems change (19) and move into a more holistic and integrated interdisciplinary paradigm, they will be challenged to find vocabulary and onto-epistemological foundations that can serve as a foundation for this study. Such a foundation needs to embrace cognitive, affective and creative epistemologies and recognize that the relational realm is an embodied intersubjective one which holds the possibility of trans-subjectivity. The realization of equine welfare will therefore benefit from greater awareness of the attentional violence arising from our inattentiveness and how we can develop our attentional proficiency as we evolve our relationships into the empathic dialogical space and beyond into the transformative space (10). It is proposed that greater attention to our injustices is inter-related across the human-equine world and will allow us to integrate concerns about multispecies justice into One Health and articulate this as One Health justice.

## Data Availability

The datasets presented in this article are not readily available because field notes are ethnographic in nature and there are therefore no “data sets” that could be made available. Requests to access the datasets should be directed to not applicable.

## Footnotes

1 Here inattention is used to indicate a form of structural violence that from societal agreements to not pay attention to those we deem less human than ourselves (81) and to challenge us to explore what is involved in cultivating a quality of attention that redresses these injustices.

2 Himself a regular visitor to Imlil.

3 “A mare and foal feeding close by had set Si Omar's horse neighing and plunging, and he, swaying a little to the plunges, may perhaps have touched it in the mouth too sharply with the bit. After a spring or two, the horse passaged and reared, and lighting, on a flat slab of rock which cropped up in the middle of the road, slipped sideways and fell with a loud crash, its shoes, in the last struggle, sending a shower of sparks into the air.” From “The Grave of the Horseman” [(45), p. 116].

4 Field Notes 12, pages 14–15 (2 May, 2014).

5 Field Notes 12, page 15 (2 May, 2014).

6 Modern bits are only available when SPANA make their once-a-month visit to Imlil and an owner presents himself and is willing to surrender their traditional bit. In many cases, there are no curb bits available. In other cases, the owner is away working on the day of the visit and never gets the opportunity to swap their bit.

7 Buber emphasizes that “both modes of speaking are necessary” for “our lives in the world benefit in practical ways because of *I-It* relations” but “developing personal wholeness requires *I-Thou* relationships” [(90, p. 16].

8 Here, “really” is understood to mean 'to know as fully and richly as possible... it invites multiplicity.

9 The *I-It* and the *I-Thou* are ever present as alternating primal “attitudes” or “ways of speaking” [(9), p. 16].

10 8,000 Moroccan Dirhams (MAD) is equivalent to about 800 €. A further 300 MAD had been paid to transport her back to Imlil.

11 Balaam's Donkey in the Bible, New International Version, Numbers 22: 21–39.

12 Ellen is an equine trainer and instructor with a particular interest in bitless riding and ethical training. She is also very proficient with a camera. As such, she was able to both help shape the training we were developing for Bella, her handlers and owners, and film this work as part of the educational approach we were developing. This approach was subsequently used as part of training programmes for muleteer teams working in the Moroccan mountain tourism industry.

14 Field Notes 21, page 21 (19th September, 2014).

15 The term “surrender” translates Buber's term “passion”.

16 Field Notes 21, page 22 (20th September, 2014).

17 This is a reference to Theodore Roosevelt's speech “Citizenship In A Republic”, delivered at the Sorbonne, in Paris, France on 23 April, 1910, in which he proposes that: “It is not the critic who counts; not the man who points out how the strong man stumbles, or where the doer of deeds could have done them better. The credit belongs to the man who is actually in the arena, whose face is marred by dust and sweat and blood; who strives valiantly; who errs, who comes short again and again, because there is no effort without error and shortcoming; but who does actually strive to do the deeds; who knows great enthusiasms, the great devotions; who spends himself in a worthy cause; who at the best knows in the end the triumph of high achievement, and who at the worst, if he fails, at least fails whilst daring greatly, so that his place shall never be with those cold and timid souls who neither know victory nor defeat.”

18 Field Notes 23, pages 1–2 (31st October, 2014).

19 Les mœurs du désert (the moral code of the desert) is the subtitle of Daumas's 19th-century classic work on Arab equestrianism in North Africa. It captures the significant contribution made by the desert environment and the warring, nomadic (and, to a lesser extent, pastoral) way of life of the tribes living there to their relationship with their horses. The contradictions listed include greed and generosity, savageness and civility. Desert life is unbearable and yet brings out the virtues of courage, loyalty, and rectitude (p. 45). It should be noted, however, that the communities I was working with, whilst having very similar practices to their nomadic cousins, are sedentary and at peace.

20 The fatalism that characterizes such societies is a significant factor in the absence of such challenges.

21 Kelly Marks proposes several useful questions to ask when problems occur, including asking yourself how you would go about creating the problem in the first place [(59), pp. 34–35].

22 See Marks [(59), pp. 47–49] for a discussion of the utility of food in training.

23 Field Notes 23, page 22 (5th November, 2014).

24 Turning away from everything that would prevent us from entering into genuine relationship with the other and toward whoever or whatever present itself to us [(9), p. 163].

25 Field Notes 25, pages 6–7 (20th December, 2014).

26 When used properly, food can create positive associations [(59), pp. 47–49 and 116–117].

27 Strictly speaking, we would take one step forwards, two back, then perhaps two forwards, without making any headway.

28 Field Notes 25, page 12 (22 December, 2014).

29 This is an example of attending to the objective and not to the other, who may be tiring, whose attention may be drifting.

30 At the foot of the U one is confronted with Source: one's Self, one's work and purpose.

31 It is the first standard factual category provided by the Oxford Dictionary of National Biography [(64), p. 21].

32 And the “bonds” that may exist between humans and equines.

33 This is the threshold of the emergent future (31), the narrow ridge on which *I-Thou* meet [(21), p. 218; (94)].

34 “Méfie-toi du mulet, même mort il te frapperait de ses fers.” [(68), p. 38).

35 Field Notes 24, p. 35 (25th November, 2014).

36 Or indeed a warning kick—for close attention to what a mule says allows you to pick up the clear messages that she offers to those willing and able to listen. Failure to heed (i.e., to fully read and register) these messages and modify one's own behavior is what precipitates the kick.

37 See Buber's description of his own childhood encounters with a horse [(95), pp. 66, 67] for his own description of this distinction, of this turning.
